# Enhanced estimation method for partial scattering functions in contrast variation small-angle neutron scattering via Gaussian process regression with prior knowledge of smoothness

**DOI:** 10.1107/S1600576725003334

**Published:** 2025-05-31

**Authors:** Ippei Obayashi, Shinya Miyajima, Kazuaki Tanaka, Koichi Mayumi

**Affiliations:** a Center for Artificial Intelligence and Mathematical Data Science, Okayama University, Japan; b Faculty of Science and Engineering, Iwate University, Japan; c Global Center for Science and Engineering, Waseda University, Japan; d Institute for Solid State Physics, University of Tokyo, Japan; Argonne National Laboratory, USA

**Keywords:** contrast variation small-angle neutron scattering, CV-SANS, partial scattering functions, multi-component systems, statistical methods, Bayesian inference, contrast variation, Gaussian process regression

## Abstract

Contrast variation small-angle neutron scattering (CV-SANS) is a powerful tool for evaluating the structure of multi-component systems. In CV-SANS, the scattering intensities *I*(*Q*) measured with different scattering contrasts are de­com­posed into partial scattering functions *S*(*Q*) of the self- and cross-correlations between components. Since the measurement has a measurement error, *S*(*Q*) must be estimated statistically from *I*(*Q*). If no prior knowledge about *S*(*Q*) is available, the least-squares method is best, and this is the most popular estimation method. However, if prior knowledge is available, the estimation can be improved using Bayesian inference in a statistically authorized way. In this paper, we propose a novel method to improve the estimation of *S*(*Q*), based on Gaussian process regression using prior knowledge about the smoothness and flatness of *S*(*Q*). We demonstrate the method using synthetic core–shell and experimental polyrotaxane SANS data.

## Abstract 2

A novel method is proposed to improve the estimation of partial scattering functions from contrast variation small-angle neutron scattering (CV-SANS) data, based on Gaussian process regression using prior knowledge about the smoothness and flatness of *S*(*Q*). The method is demonstrated using computational core–shell and experimental polyrotaxane SANS data.

## 1. Introduction

Small-angle neutron scattering with contrast variation (CV-SANS) has been used to observe separately the nano-scale structure of each component in a multi-component system, such as polymer/nanoparticle mixtures (Endo *et al.*, 2008 ▸; Takenaka *et al.*, 2009 ▸), copolymer micelles (Richter *et al.*, 1997 ▸), mechanically interlocked supramolecules (Mayumi *et al.*, 2009 ▸; Endo *et al.*, 2011 ▸), protein complexes (Jeffries *et al.*, 2016 ▸) and biological membranes (Nickels *et al.*, 2017 ▸). In the case of *p*-component systems, the SANS intensity *I* is a sum of partial scattering functions *S*_*ij*_ (Endo, 2006 ▸),
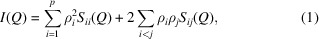
where *Q* is the magnitude of the scattering vector, ρ_*i*_ is the scattering length density of the *i*th component, *S*_*ii*_(*Q*) is a self-term corresponding to the structure of the *i*th component, and *S*_*ij*_(*Q*) is a cross-term representing the correlation between the *i*th and *j*th components. Under the assumption of incompressibility, equation (1) can be reduced to the following (Endo, 2006 ▸): 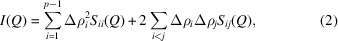
where Δρ_*i*_ = ρ_*i*_ − ρ_*p*_. In the following, we assume that (*p* − 1) solutes (*i* = 1,…, *p* − 1) are dissolved in a solvent (*i* = *p*). Then, Δρ_*i*_ is the scattering length density difference between the *i*th solute and the solvent.

Using relationship (2), we can determine the partial scattering functions by measuring *I*(*Q*) of *N* samples [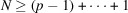 = 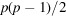] with different scattering contrasts [(Δ_1_ρ_1_,…, Δ_1_ρ_*p*_),…, (Δ_*N*_ρ_1_,…, Δ_*N*_ρ_*p*_)]. The following shows the relationship between samples *I*_1_(*Q*),…, *I*_*N*_(*Q*) and the partial scattering functions: 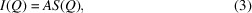
where 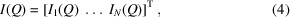

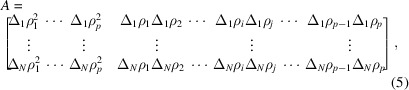

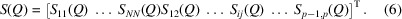


Experimentally obtained *I*_*i*_(*Q*) have errors that must be treated appropriately. We introduce an error term to (3) as follows: 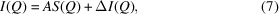
where Δ*I*_1_(*Q*),…, Δ*I*_*N*_(*Q*) are the errors in each experiment, and 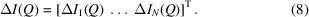


A popular error treatment method is least squares. We solve the following least-squares problem to find the appropriate *S*(*Q*) from measurements *I*(*Q*): 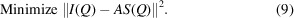
The Gauss–Markov theorem ensures the validity of the least-squares method. The theorem states that the solution has the lowest sampling variance within the class of linear unbiased estimators if the errors are uncorrelated and have equal variances. Even if the errors do not have equal variances, the Gauss–Markov theorem is valid with a modification using weighted least squares {in our setting, ∥Σ^−1^[*I*(*Q*) − *AS*(*Q*)]∥^2^ is minimized instead of ∥I(*Q*) − *AS*(*Q*)∥^2^, where Σ is the covariance matrix}. The (weighted) least-squares solution is the best among the unbiased estimators.

However, scientists have found that introducing bias improves the estimator in various cases. Introducing bias is equivalent to introducing prior knowledge into the estimation using the Bayesian framework (Gelman *et al.*, 2004 ▸). Bayesian inference has already been applied to CV-SANS data to evaluate the errors of the estimated *S*(*Q*) in our previous paper (Mayumi *et al.*, 2025 ▸). In that paper, we used a non-informative prior, which means that we made few assumptions about the partial scattering functions. In the situation considered here, we have prior knowledge about the smoothness and flatness of the partial scattering functions *S*(*Q*) along the **Q** direction, which has not yet been used in the estimation of partial scattering functions by Bayesian inference. The information about the smoothness and flatness of the partial scattering functions drastically improves the estimation of the partial scattering functions. Gaussian process regression (MacKay, 1998 ▸; Rasmussen & Williams, 2005 ▸), a type of Bayesian inference, can be used for our purposes. This paper presents a new method for estimating partial scattering functions from scattering intensities. It modifies the Gaussian process regression method.

Gaussian process regression is a Bayesian approach for estimating functions or curves from given data. It utilizes the Gaussian process, a probabilistic distribution of a set of functions. The treatment of such a probabilistic distribution is generally difficult, but Gaussian process regression provides a smart solution. The Gaussian process is used for geostatistics (kriging in that field), computer experiments and machine learning. See Section 2.8 of the work of Rasmussen & Williams (2005 ▸) for a brief history and related studies on Gaussian process regression.

In our setting, we encode the prior knowledge about partial scattering functions into a prior distribution using a kernel function, and we calculate the posterior distribution of the partial scattering functions from the prior distribution and experimentally obtained data. The posterior distribution gives us the most certain estimators and their certainty in the form of a multivariate Gaussian distribution. We can obtain error bars from the posterior distribution.

The advantage of the proposed method is that it allows us to introduce prior knowledge about the smoothness and flatness of the partial scattering functions in a statistically authorized way. Such prior knowledge improves the estimation of partial scattering functions without additional experiments. We could also smooth the data by applying a Gaussian filter, but it is unclear how one can properly determine error bars when using it. The proposed method gives statistically reasonable error bars.

To apply the proposed method to CV-SANS data, we need to select some kernel function parameters. Another advantage of our proposed method is that it provides a systematic way of choosing the parameters from the viewpoint of Bayesian statistics. Three approaches, called subjective Bayesian approach, subjective Bayesian approach using a hyper-prior and empirical Bayesian approach, are proposed in this paper.

The proposed method is slightly modified from standard Gaussian process regression. Gaussian process regression is usually used to estimate a function from noisy samples of the function and it can be used to estimate noise-reduced intensity functions. In contrast, the proposed method directly estimates partial scattering functions by modifying the Gaussian process regression method.

In the field of scattering experiments, Bayesian inference has been increasingly applied to enhance data analysis, including model parameter estimation (Antonov *et al.*, 2016 ▸; Larsen *et al.*, 2018 ▸; Hayashi *et al.*, 2023 ▸), model selection (Hayashi *et al.*, 2024 ▸) and estimation of pair distance distribution functions (PDDFs) through inverse Fourier tranformation (IFT) (Hansen, 2000 ▸; Larsen & Pedersen, 2021 ▸). In the research on model parameter estimation (Antonov *et al.*, 2016 ▸; Larsen *et al.*, 2018 ▸; Hayashi *et al.*, 2023 ▸), model parameters have been directly estimated as probability distributions from small-angle scattering (SAS) data, providing not only accurate estimates but also a quantitative assessment of estimation reliability. The methods used focus on parameter estimation for predetermined models. Recent research by Hayashi *et al.* (2024 ▸) added a model selection ability to the method. Research on the combinations of IFT and Bayesian inference (Hansen, 2000 ▸; Larsen & Pedersen, 2021 ▸) proposed methods to estimate PDDFs from SAS data in a Bayesian way. In these methods, a PDDF is represented by a weighted sum of a suitable set of basis functions and the weights are adjusted in a Bayesian way.

Our research in this paper differs from previous work regarding its aim and statistical methodology. Our research aims to estimate the partial scattering functions from CV-SANS data, and Bayesian inference has only been applied in our previous research (Mayumi *et al.*, 2025 ▸). The more important novelty of our research is using Gaussian process regression. To the best of our knowledge, Gaussian process regression has not been used in previous research on SAS data analysis. Gaussian process regression enables us to represent our assumptions about smoothness and flatness in a statistically reasonable way. As shown below, the assumptions improve the estimated partial scattering functions compared with the conventional (weighted) least-squares method. The basis function method can also represent such assumptions by selecting basis functions and penalizing weights through first and second derivatives, as proposed in the previous PDDF estimation research (Hansen, 2000 ▸; Larsen & Pedersen, 2021 ▸). We need to design the basis functions and penalty *ad hoc* for the basis function approach. Gaussian process regression is an extension of the basis function method, as shown in ch. 2 of the textbook by Rasmussen & Williams (2005 ▸). Gaussian process regression provides a sophisticated way of generalizing the basis function approach by designing kernel functions. For example, it can naturally treat infinite-dimensional function space and represent assumptions such as smoothness. Various kernel design techniques are available to extend our proposed method: see ch. 4 of Rasmussen & Williams (2005 ▸).

**Figure 1:**
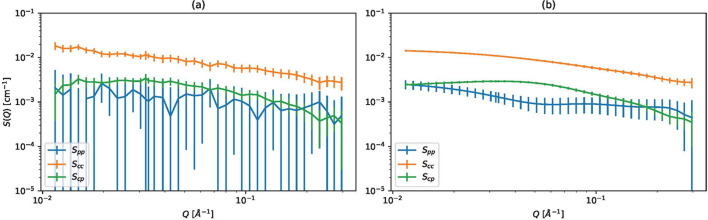
Demonstration of the proposed method. Section 3.2 discusses the results in more detail. (*a*) Estimated partial scattering functions and their error bars using the method proposed by Mayumi *et al.* (2025 ▸). (*b*) Estimated partial scattering functions using our proposed method with the Matérn 5/2 kernel and α = 0.01, *l* = 0.2.

Fig. 1 ▸ demonstrates the power of our method. From the same experimentally observed intensity functions, partial scattering functions are estimated using (*a*) the previous research method and (*b*) the proposed method. The proposed method gives better results with small error bars. The mechanism to improve the results uses near-*Q* intensity data to estimate *S*(*Q*) through the prior distribution. Our proposed method uses more information than least squares. The details of the results will be discussed in Section 3.

The advantages of the proposed method are summarized as follows:

(i) The method enhances the estimation of the partial scattering functions with almost no additional cost using the smoothness and flatness assumptions.

(ii) The method gives statistically reasonable error bars.

(iii) The number of tuning parameters of the method is small.

(iv) How to select parameters in a systematic way is also proposed.

The proposed method will help CV-SANS users. Longer experiments are needed to improve the accuracy of the observations, but neutron scattering experiments are expensive. Our proposed method from mathematical statistics will reduce the cost of CV-SANS experiments.

## 2. Methods

### 2.1. Partial scattering function estimation method

We first introduce some notation to describe the method. *Q*_1_,…, *Q*_*M*_ are the magnitudes of the scattering vectors of the experiment. That is, *I*_*n*_(*Q*_*m*_) for *n* = 1,…, *N* and *m* = 1,…, *M* are experimentally obtained scattering intensities. Correspondingly, the error terms are described as follows for *m* = 1,…, *M*: 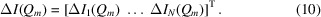
The index of *S* is changed as follows to simplify the explanation: 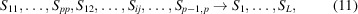
where *L* = *p*(*p* − 1)/2 is the number of partial scattering functions of self- and cross-correlations. Correspondingly, we express the matrix *A* as follows: 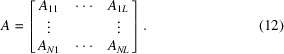


For statistical estimation, we need to make some assumptions about errors. We assume that the errors Δ*I*_*n*_(*Q*_*m*_) are statistically independent and the probabilistic distribution of the errors is a Gaussian distribution. We also assume that the variance of each Gaussian distribution is known; we write this as 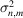. We do not assume equal variance.

Using Gaussian process regression, we introduce a kernel function to represent prior knowledge about the smoothness and flatness of *S*_ℓ_(*Q*). The kernel function 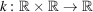 satisfies the following conditions:

(i) *k* is symmetric. That is, *k*(*P*, *Q*) = *k*(*Q*, *P*) for any *P* and *Q*.

(ii) *k* is positive definite. That is, 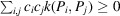 for any *c*_*i*_ ∈ 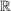, *P*_*i*_ ∈ 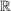.

Using the kernel function *k*, we introduce a prior distribution on *S*_ℓ_(*Q*_*m*_) for ℓ = 1,…, *L*, *m* = 1,…, *M* under the following assumptions:

(i) The prior distribution is a multivariate Gaussian distribution whose mean is zero.

(ii) For different ℓ and ℓ′, *S*_ℓ_(*Q*_*m*_) and 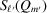 are statistically independent for every *m*, *m*′ = 1,…, *M*.

(iii) For a common ℓ, 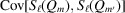 = 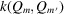 for each *m*, *m*′ = 1,…, *M*.

Under the above assumptions, the following horizontally reordered *LM*-dimensional vector 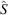 has a prior distribution 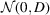: 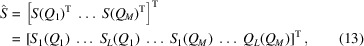

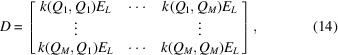
where *E*_*L*_ is an *L* × *L* identity matrix. The meaning of the prior distribution is as follows:

(i) The assumption of a multivariate Gaussian distribution is based on theoretical and computational reasons. Theoretically, this assumption ensures the existence of a stochastic process from the viewpoint of probability theory. Practically, this assumption enables us to compute the posterior distribution only using linear algebra.

(ii) The independence between *S*_ℓ_(*Q*) and 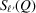 for different ℓ, ℓ′ means that we have no special knowledge about the relationship between two different partial scattering functions.

(iii) The covariance between *S*_ℓ_(*Q*_*m*_) for *m* = 1,…, *M* represents the prior knowledge about the partial scattering function *S*_ℓ_(*Q*), and the choice of the kernel function determines the smoothness and flatness.

Therefore, the choice of kernel function is important. We will discuss the effect of the kernel function later.

Now, *I*(*Q*_*m*_) and Δ*I*(*Q*_*m*_) are reordered as follows to match 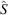: 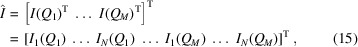

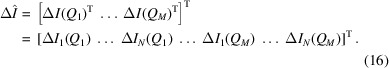
Then (7) can be represented by the following with the above assumptions: 
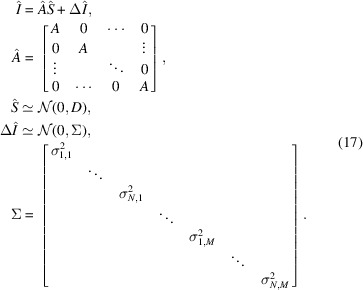
From the standard formula for a linear Bayesian estimation, the posterior of 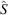 is the following multivariate Gaussian distribution: 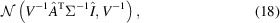
where 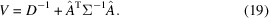
We interpret the posterior as follows:

(i) The elements of the mean vector 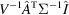 are the most certain estimators of the partial scattering functions *S*_ℓ_(*Q*_*m*_).

(ii) The diagonal elements of the covariance matrix *V*^−1^ represent the uncertainty of the estimators in the form of variances.

(iii) The off-diagonal elements of the covariance matrix *V*^−1^ represent the covariance between *S*_ℓ_(*Q*_*m*_) and 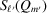.

That is, the mean vector gives the estimators of the partial scattering functions, and the square roots of the diagonal elements of the covariance matrix give the estimators’ error bars.

### 2.2. Kernel functions

Many kernel functions are known and are in use. In this paper, we consider the following two representative kernels.

(i) The Gaussian kernel: 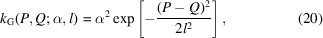
where α, *l* > 0 are parameters. When using the Gaussian kernel in Gaussian process regression, it is assumed that the estimated functions are infinitely differentiable and very smooth.

(ii) The Matérn kernel: 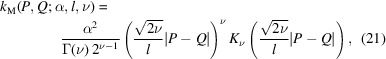
where α, *l* and ν are parameters, Γ is the gamma function and *K*_ν_ is a modified Bessel function. ν controls the differentiability of the estimated functions; the estimated functions are 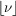 times differentiable, where 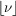 is the floor of ν. Usually, ν = 1/2, 3/2 and 5/2 are used, but in this paper we only use 3/2 and 5/2.

The above kernels allow us to introduce different smoothnesses to the estimated functions. Therefore, these kernels are suitable for our purpose since we want to introduce prior knowledge about the smoothness of the partial scattering functions. It is worth comparing these three kernels since they introduce different smoothnesses. We do not consider the Matérn 1/2 kernel in this paper since it requires the assumption that the estimated functions are not differentiable, which is not suitable for our purposes.

The selection of the parameters of the kernel functions is also important. The above two kernels have two common parameters, α and *l*. For both kernels, the parameter α controls the total effect of the kernel. As α becomes larger, the effect becomes weaker. For both kernels, the parameter *l* controls the flatness of the estimated results. As *l* becomes larger, the estimated partial scattering functions become flatter; that is, the estimated functions become less jagged, less bumpy and more monotonic. If the scale parameters become extremely large, the estimated functions become completely flat. This behavior occurs because when *l* is large *I*(*Q*) with a wider range of *Q* is used to estimate *S*(*Q*_*m*_) for a single *Q*_*m*_. *l* is called the *scaling parameter*. The next subsection discusses how to select parameters. The changes in the predictions when the parameters are changed will be discussed in Section 3.

We also introduce a white kernel, which can be used to improve the above kernels. The definition of the white kernel is as follows: 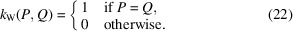
The white kernel is used by adding τ^2^*k*_W_ to another kernel with a very small positive τ. Theoretically, the addition of the white kernel requires assuming the uncertainty in *S*(*Q*), which is not included in the model introduced in Section 2.1, since the white kernel represents white noise of strength τ. Practically, the white kernel stabilizes the results. The effect of the white kernel is discussed in Section 3.1.

### 2.3. Selection of parameters

We need to select kernel parameters to apply our methods to SANS data. As shown in the later sections, we can change the estimated results by changing the parameters. This fact means that we can intentionally control the results, which may undermine the validity of the scientific reasoning. To avoid this problem, we require a systematic parameter selection method.

The above problem is known as model selection in statistics. In this paper, we also introduce subjective and empirical Bayesian approaches. Related to these approaches, we introduce a marginal likelihood from the textbook of Rasmussen & Williams (2005 ▸) on Gaussian process regression

#### 2.3.1. Subjective Bayesian approach

One way to select parameters is to refer to previous research and preliminary real and numerical experiments. Choosing parameters after seeing the results changes the prior knowledge after the experiments. To avoid this problem, we use experimental knowledge from literature research and preliminary experiments as prior knowledge. Of course, this paper can itself be available as part of the prior knowledge. Examining parameter ranges and using common trends in those results are good approaches, and such findings do not depend on the parameter choice.

#### 2.3.2. Subjective Bayesian approach using a hyper-prior

Bayesian statistics provides a sophisticated treatment of parameter ranges. The first step is representing the parameter ranges obtained from prior knowledge in a probabilistic distribution *p*(θ), where θ is a vector of all kernel parameters. For the Gaussian and Matérn kernels, θ is (α, *l*). We regard the distribution as a prior on the parameters, and we can compute the posterior on the parameters 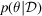 as follows using Bayes’ theorem: 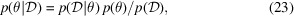
where θ represents all the kernel parameters, 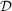 represents all the experimentally observed data and 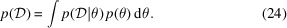

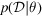
 is called the *marginal likelihood* and has the following explicit expression (Rasmussen & Williams, 2005 ▸): 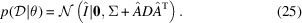
We note that the matrix *D* depends on θ since *D* is computed from a kernel function.

The prior distribution *p*(θ) is called the *hyper-prior*, and we need to determine the hyper-prior from prior knowledge, such as previous research and preliminary experiments.

We can use the hyper-posterior calculated from the hyper-prior in the following ways:

(i) The estimated distribution of the partial scattering functions is averaged by the hyper-posterior.

(ii) θ which maximizes 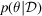 is adopted.

The latter is called the MAP (maximize *a posteriori*) estimation. Since computing the average is often difficult, MAP estimation is often used. The Laplace approximation is also used to address the difficulty (Williams & Barber, 1998 ▸; Bishop, 2007 ▸).

The selection of the hyper-prior depends on our prior knowledge. If we have scant prior knowledge, we often use weakly informative priors. One typical distribution of weakly informative priors is the Gaussian distribution with large variances. For the Gaussian and Matérn cases, since the parameters α and *l* should be positive, the log-normal distribution or gamma distribution is also suitable for the weakly informative hyper-prior.

When the kernel parameters are estimated using MAP estimation, we do not need to compute (24) since 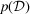 does not depend on θ. All we have to do is maximize the following function with respect to θ: 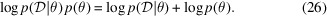
The log marginal likelihood 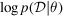 has the following explicit formula from (25): 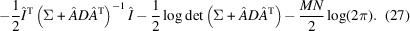


#### 2.3.3. Empirical Bayesian approach

When the kernel parameters are estimated using MAP estimation and we use the Gaussian distribution as a weakly informative hyper-prior, (26) can be expressed as follows: 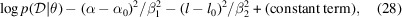
where α_0_ and *l*_0_ are the center of the hyper-prior and β_1_, β_2_ > 0 are standard deviations of the hyper-prior. Since β_1_ and β_2_ represent the uncertainty in the kernel parameters, β_1_ and β_2_ should be large. When β_1_, β_2_ → ∞, the above goes to 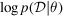. This means that this log marginal likelihood is available to evaluate the suitability of the kernel parameters. We select kernel parameters that maximize the log marginal likelihood (27). This approach is called the empirical Bayesian approach.

#### 2.3.4. Comparison of the three approaches

We have introduced three approaches above for selecting kernel parameters. The first and second approaches are often called subjective Bayes and the third approach is called empirical Bayes. We must consider which approach is best for our purpose. The first approach (Section 2.3.1) works well if we have sufficient prior knowledge. If the prior knowledge is scant, the second and third approaches (Sections 2.3.2 and 2.3.3) are good.

Whichever approach we choose, it is important to decide on it before the analysis. We should not subsequently change the method, to avoid intentionally controlling the estimated results. This paper uses the log marginal likelihood since it is easy to compute.

### 2.4. Computational data of a core–shell sphere

**Figure 2:**
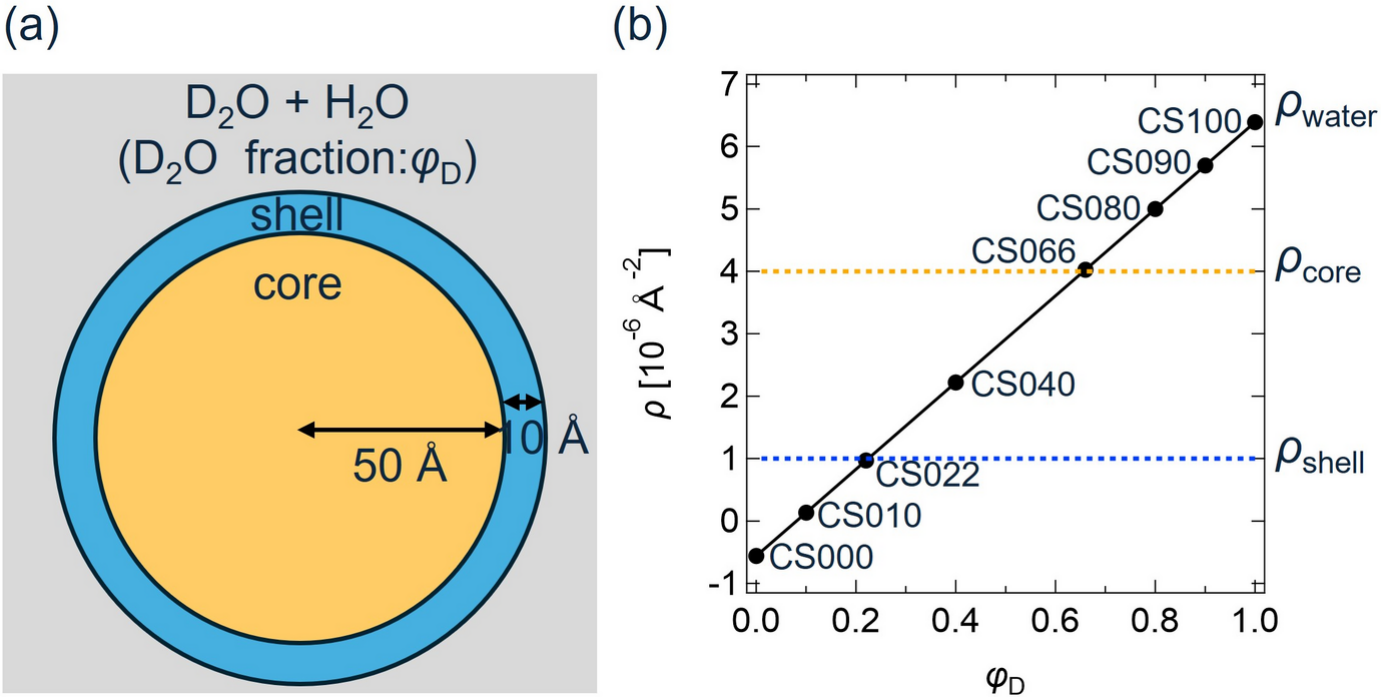
(*a*) Schematic illustration of a core–shell sphere dispersed in a D_2_O/H_2_O mixture. (*b*) Scattering length densities of the core (ρ_core_), shell(ρ_shell_) and solvent(ρ_water_) plotted against the D_2_O fraction of the solvent ϕ_D_. Reproduced from Mayumi *et al.* (2025 ▸).

**Figure 3:**
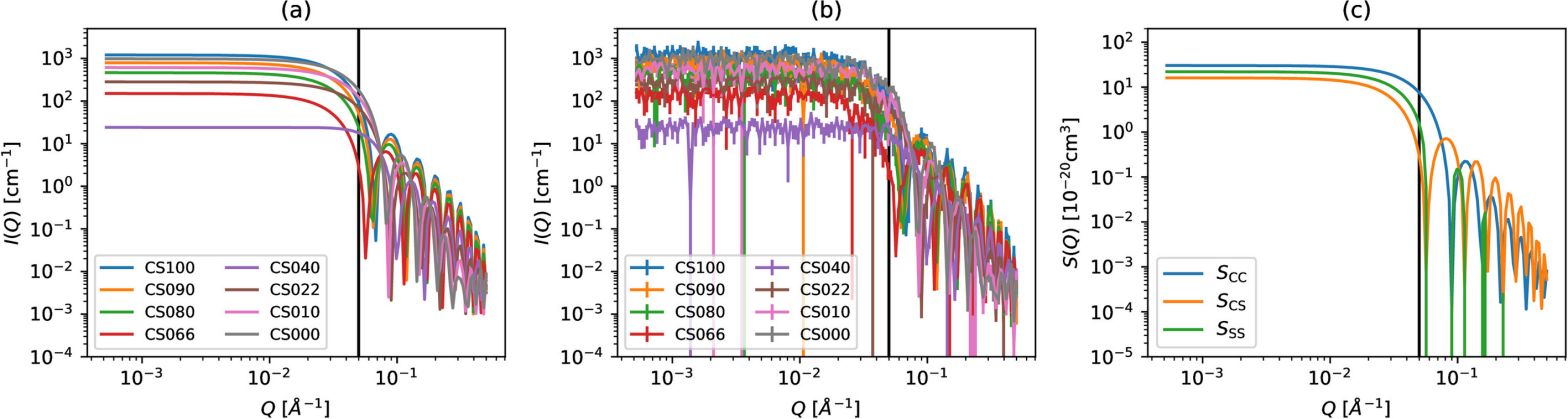
Synthetic scattering intensities of core–shell spheres. The vertical lines indicate the upper bound of CORE-SHELL-A data introduced in Section 3.1. (*a*) Scattering intensities computed by the core–shell model. (*b*) Scattering intensities after noise is added. The error bars indicate σ_*n*,*m*_. (*c*) Expected partial scattering functions.

To verify the validity of the proposed method, we applied it to computational SANS data (Mayumi *et al.*, 2025 ▸). We used the ‘core–shell sphere’ model of the *SASview* software (https://www.sasview.org/) to compute the scattering intensities of core–shell spheres dispersed in D_2_O/H_2_O mixtures with different D_2_O fractions [Fig. 2 ▸(*a*)]. The core radius and shell thickness were 50 and 10 Å, respectively. While the scattering length densities of the core and shell were fixed at 4.0 and 1.0 × 10^−6^ Å^−2^, the scattering length density of the solvent was changed with the D_2_O fraction ϕ_D_ as follows (Endo *et al.*, 2008 ▸): 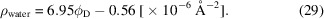
Here, the core–shell samples with ϕ_D_ = 1.0, 0.90, 0.80, 0.66, 0.40, 0.22, 0.10 and 0.0 are named CS100, CS090, CS080, CS066, CS040, CS022, CS010 and CS000, respectively [Fig. 2 ▸(*b*)]. After computing the scattering intensities, multiplicative noise was added as follows: 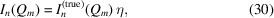
where 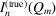 is the scattering intensity computed from the core–shell model and η is a random number taken from 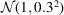. The standard deviation σ_*n*,*m*_ was set to 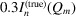. According to equation (2), *I*(*Q*) of the core–shell sphere is described as 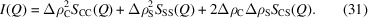
Here, *S*_CC_(*Q*) is the self-term of the core, *S*_SS_(*Q*) is the self-term of the shell and *S*_CS_(*Q*) is the cross-term between the core and the shell. Fig. 3 ▸ shows the computed scattering intensities and expected partial scattering functions.

In this numerical experiment, we used two types of data. The first only includes data in the *Q* < 0.05 Å^−1^ range, called CORE-SHELL-A. The second includes all the data and is called CORE-SHELL-B. CORE-SHELL-A are the data to the left of the vertical lines in Fig. 3 ▸. By comparing the two results, we examined the effect of singular data within the high-*Q* range.

### 2.5. Experimental data for polyrotaxane solutions

**Figure 4:**
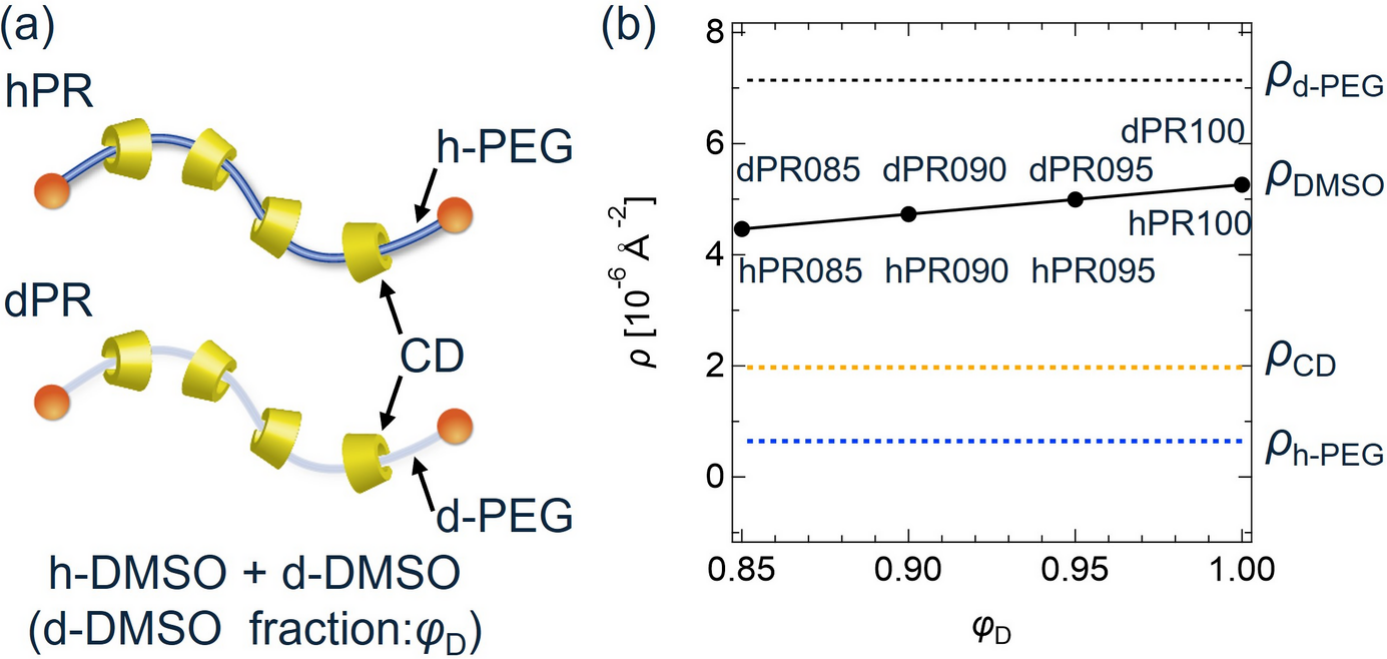
(*a*) Schematic illustration of a polyrotaxane solution dissolved in a d-DMSO/h-DMSO mixture. (*b*) Scattering length densities of h-PEG (ρ_h-PEG_), d-PEG(ρ_d-PEG_), CD(ρ_CD_) and solvent (ρ_DMSO_) plotted against d-DMSO fraction of the solvent ϕ_D_. Reproduced from Mayumi *et al.* (2025 ▸).

This method was also applied to experimental CV-SANS data of polyrotaxane (PR) solutions. PR is a mechanically interlocked supramolecular assembly in which ring molecules are threaded onto a linear polymer chain. We used CV-SANS data of PR consisting of polyethylene glycol (PEG) and α-cyclodextrins (CDs), as reported in our previous papers (Mayumi *et al.*, 2009 ▸; Mayumi *et al.*, 2025 ▸). For the CV-SANS measurements, we prepared hPR with hydrogenated (h-) poly-ethylene glycol (PEG) and dPR containing deuterated (d-) PEG [Fig. 4 ▸(*a*)]. The scattering length densities ρ of h-PEG, d-PEG and CD were 0.65 × 10^6^, 7.1 × 10^6^ and 2.0 × 10^6^ Å^−2^, respectively. h-PR and d-PR were dissolved in mixed solvents of hydrogenated dimethyl sulfoxide (h-DMSO) and deuterated DMSO (d-DMSO). The PR volume fraction was 8%. The volume fractions of d-DMSO in the solvents, ϕ_D_, were 1.0, 0.95, 0.90 and 0.85 to change the scattering contrast. The scattering length densities of the solvents were 5.3 × 10^6^, 5.0 × 10^6^, 4.7 × 10^6^ and 4.5 × 10^6^ Å^−2^, respectively. The hPR and dPR solutions with different ϕ_D_ are named hPR100, hPR095, hPR090, hPR085, dPR100, dPR095, dPR090 and dPR085, as shown in Fig. 4 ▸(*b*).

The SANS measurements for the PR solutions were performed at 298 K using the SANS-U diffractometer of the Institute for Solid State Physics at the University of Tokyo, located in the JRR-3 research reactor of the Japan Atomic Energy Agency in Tokai, Japan. The incident beam wavelength was 7.0 Å and the wavelength distribution was 10%. The sample-to-detector distance was 1 or 4 m. Scattered neutrons were collected with a two-dimensional detector and then the necessary corrections were made, such as air and cell scattering subtractions. After these corrections, the scattered intensity was normalized to the absolute intensity using a standard polyethylene film with known absolute scattering intensity. The two-dimensional intensity data were circularly averaged and the incoherent scattering was subtracted. The averaged scattering intensities *I* were plotted against the magnitude of the scattering vector *Q*. The error bars in *I*(*Q*) were given by Δ*I* = ±σ, where σ represents the standard deviation of the circular averaging.

## 3. Results and discussion

In this section, we apply the proposed method to synthetic data and experimental data. We also compare the results of the weighted least squares, since the errors do not have equal variance. The error bars of weighted least-squares solutions were computed by the statistical error estimation proposed by Mayumi *et al.* (2025 ▸).

### 3.1. Application to synthetic data of a core–shell sphere

To verify the validity of the proposed method, we first applied it to synthetic data for the core–shell sphere introduced in Section 2.4.

**Figure 5:**
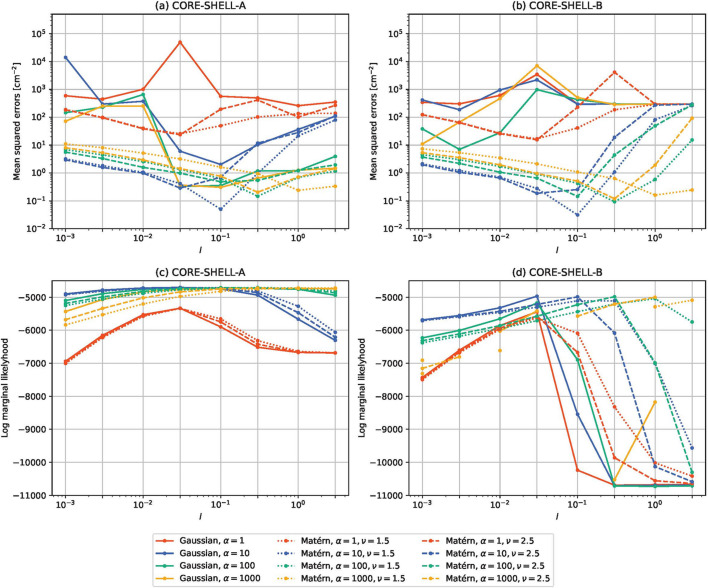
Mean squared errors and log marginal likelihoods for core–shell data with τ = 0. The mean squared errors were calculated using equation (32). (*a*) Mean squared errors between the estimated and expected partial scattering functions for CORE-SHELL-A. (*b*) Mean squared errors between the estimated and expected partial scattering functions for CORE-SHELL-B. (*c*) Log marginal likelihoods of the estimated partial scattering functions for CORE-SHELL-A. (*d*) Log marginal likelihoods of the estimated partial scattering functions for CORE-SHELL-B.

**Figure 7:**
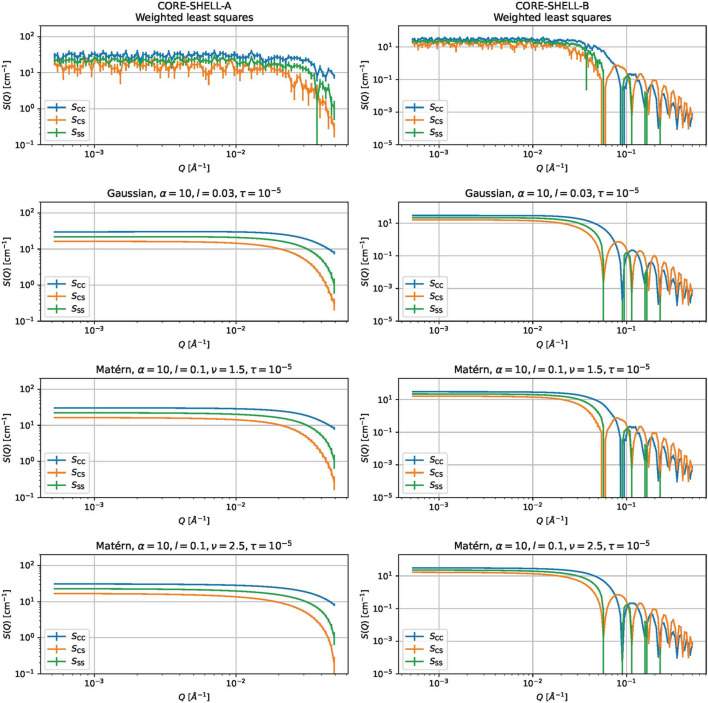
Estimated partial scattering functions for (left-hand column) CORE-SHELL-A and (right-hand column) CORE-SHELL-B. The rows are as follows, from top to bottom: results by weighted least squares, and results by the proposed methods using a Gaussian kernel (α = 10, *l* = 0.3, τ = 10^−5^), using the Matérn 3/2 kernel (α = 10, *l* = 0.1) and using the Matérn 5/2 kernel (α = 10, *l* = 0.1).

**Figure 8:**
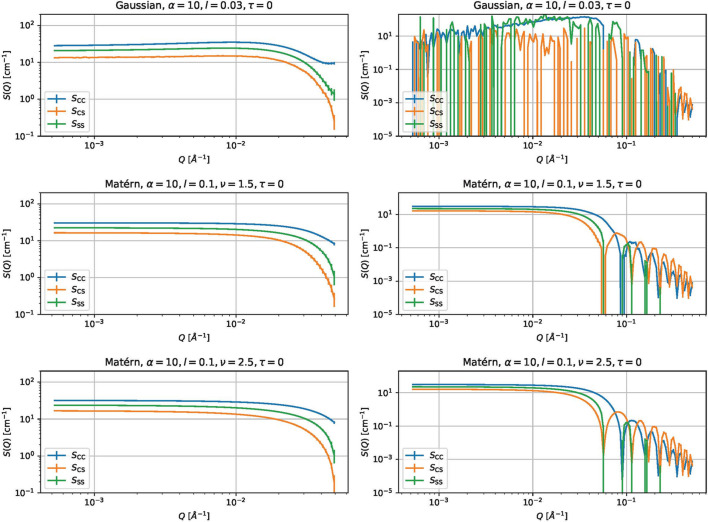
Estimated partial scattering functions for CORE-SHELL-A and CORE-SHELL-B data using the same kernels and parameters as Fig. 7 but with τ = 0.

We conducted a comprehensive parameter search and we experimented with all combinations of the following kernels and parameters: 
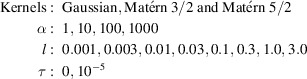
Figs. 5 ▸ and 6 ▸ show the results for τ = 0 and τ = 10^−5^, respectively. The mean squared errors between the estimated and expected partial scattering functions are shown in the top row and log marginal likelihoods are shown in the bottom row. The mean squared errors were calculated as follows: 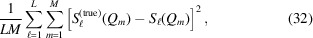
where 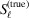 is the true (expected) partial scattering function and *S*_ℓ_(*Q*_*m*_) is the estimated partial scattering function. Fig. 7 ▸ shows some estimated partial scattering functions with good scores. Fig. 8 ▸ shows the estimated gamma with the same kernels and parameters but with τ = 0.

From the results (Figs. 5 ▸–8 ▸), we find the following:

(i) For the Matérn kernels, the effect of τ is small. In contrast, adding a small white kernel improves the results for the Gaussian kernel.

(ii) Despite this, the log marginal likelihoods for the Gaussian kernel do not depend strongly on τ.

(iii) When *l* is varied and the other parameters are fixed, the value of *l* that minimizes the mean squared error and the value that maximizes the log marginal likelihood are roughly the same, but the latter tends to be larger.

The first fact is probably due to the smoothness determined by the choice of the kernel. The Gaussian kernel introduces much more smoothness than the Matérn kernels and the estimated results are more strongly affected by singular data. The white kernel probably suppresses the excessive smoothness introduced by the Gaussian kernel.

From the above facts, we infer the following:

(i) When using the Gaussian kernel for our proposed method, adding a small white kernel to the Gaussian kernel is important.

(ii) The log marginal likelihood is useful for selecting *l*, but it may be safe to make *l* a bit smaller than the value suggested by the log marginal likelihood.

In the following, a white kernel with τ = 10^−5^ is always added to the main kernel.

**Figure 9:**
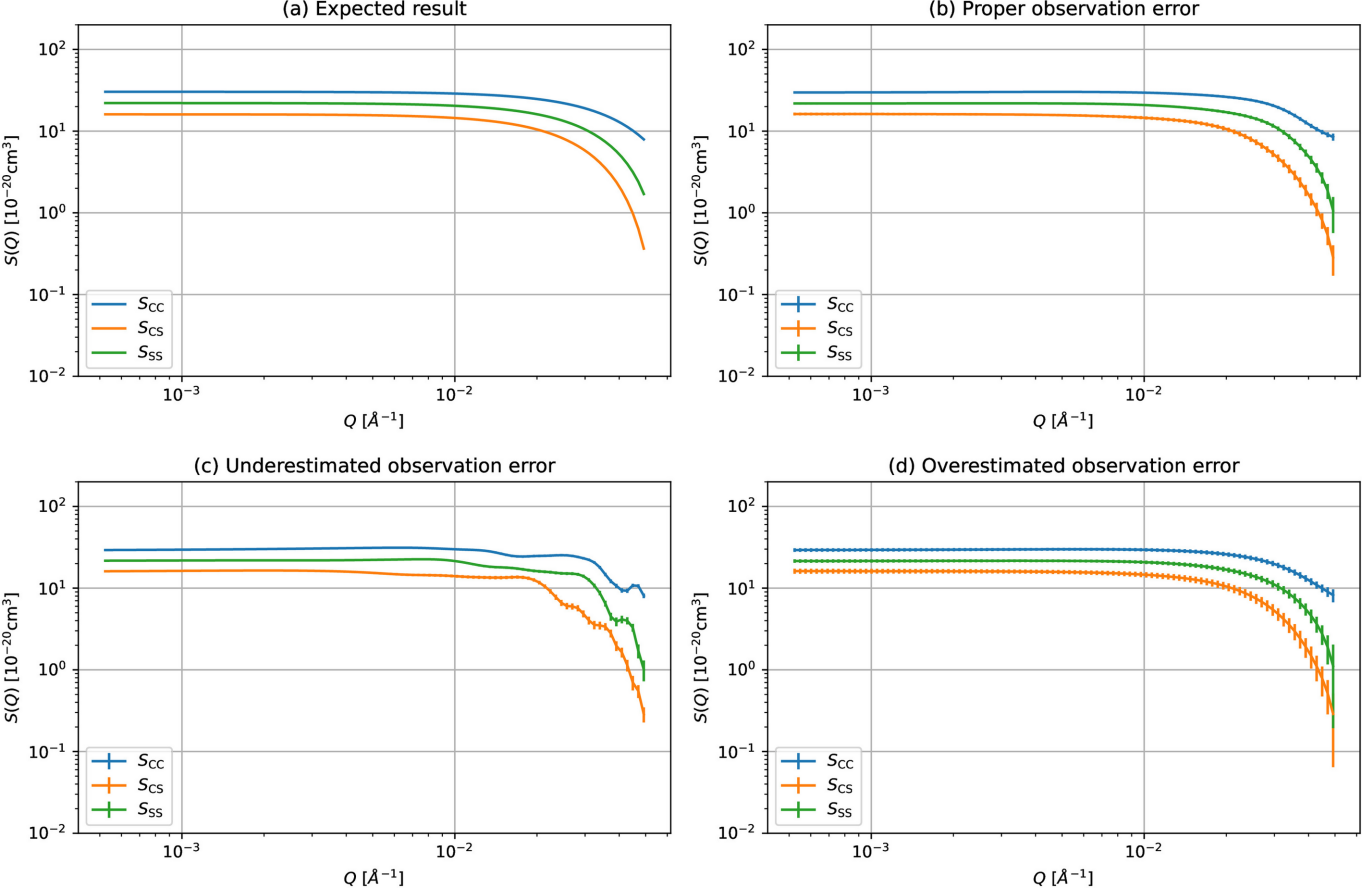
Estimated partial scattering functions and their error bars for different observation errors. (*a*) Expected partial scattering functions. (*b*) Estimated partial scattering functions for CORE-SHELL-A data. (*c*) Estimated partial scattering functions for CORE-SHELL-A data with halved observation errors. (*d*) Estimated partial scattering functions for CORE-SHELL-A data with doubled observation errors.

We also investigate the effect of underestimating and overestimating the observation errors. We applied the proposed method to the CORE-SHELL-A data, except for the points where the standard deviations σ_*n*,*m*_ were halved or doubled, using the Matérn 5/2 kernel. The kernel parameters are selected using marginal likelihoods. Fig. 9 ▸ shows the results. From the figure, we can determine the following two points:

(i) When the observation error is underestimated, the estimated partial scattering functions become wavy. This is because the underestimated error makes the observed data appear more reliable than they truly are.

(ii) When the observation error is overestimated, the estimated partial scattering functions look reasonable, but the error bars become larger than when the observation error is properly given.

From the above points, we conclude that the observation error should be appropriately estimated, but overestimation is better than underestimation.

### 3.2. Application to polyrotaxane SANS data

Next, we applied the proposed method to polyrotaxane SANS data. Since we had no ground truth for the experimental data, we could not measure the errors quantitatively as we did in Section 3.1. Therefore, we evaluated the estimations qualitatively.

**Figure 10:**
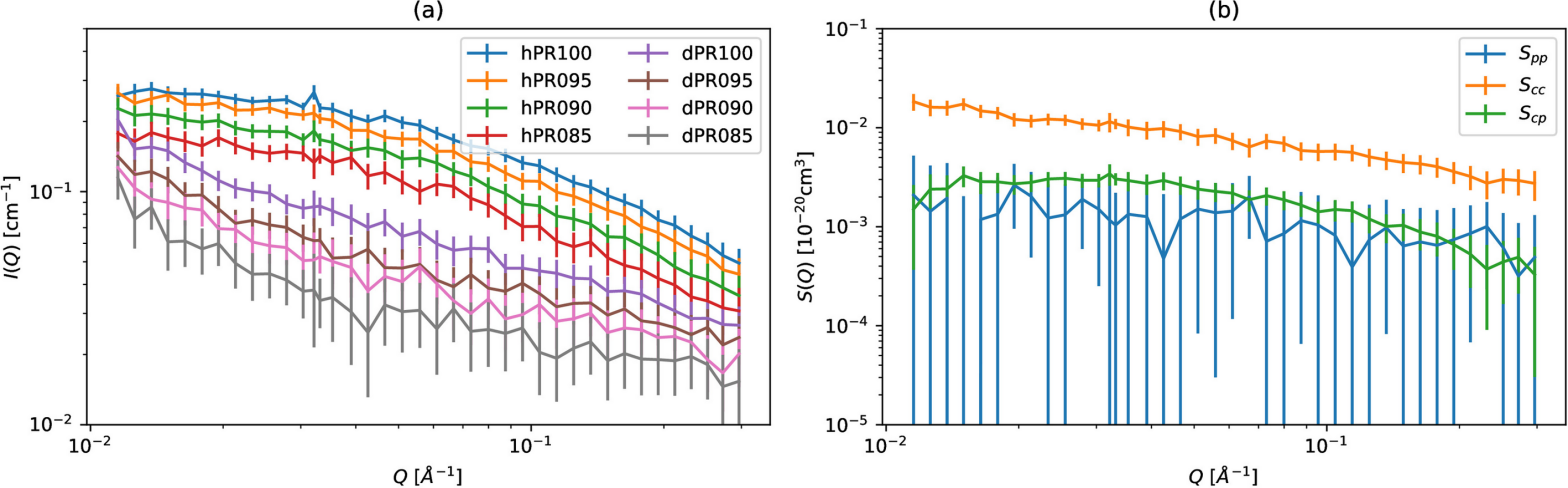
Polyrotaxane SANS data. (*a*) Scattering intensities for polyrotaxane SANS data. (*b*) Partial scattering functions estimated by weighted least squares.

Fig. 10 ▸(*a*) shows the experimentally observed scattering intensities and Fig. 10 ▸(*b*) shows the partial scattering functions estimated by weighted least squares. The error bars of *S*_CC_ and *S*_CP_ in Fig. 10 ▸(*b*) are relatively small, but the error bars of *S*_PP_ are quite large. The large error bars mean the estimated *S*_PP_ is unreliable.

**Figure 11:**
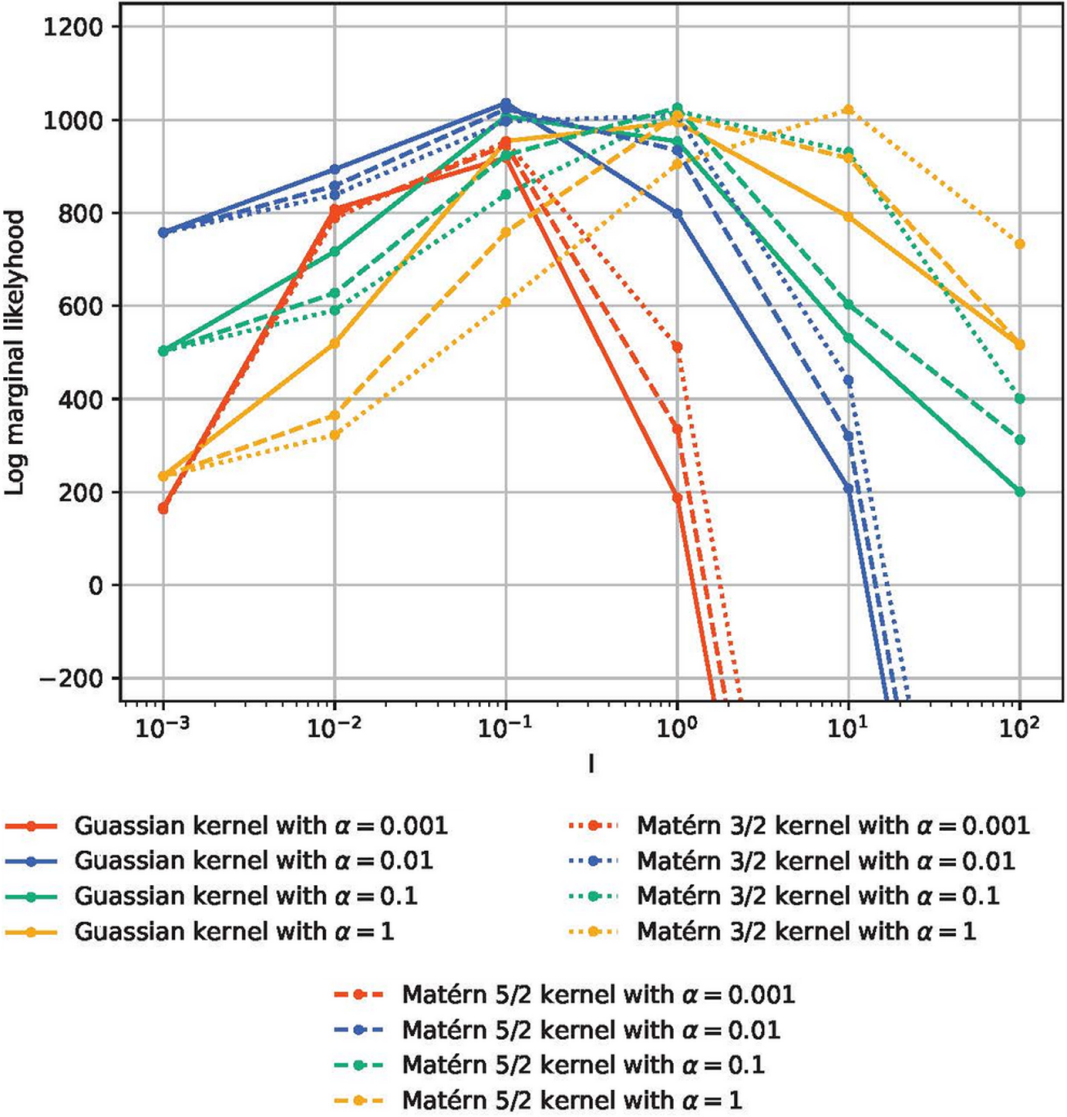
Log marginal likelihoods for polyrotaxane SANS dat.a

We conducted a comprehensive parameter search for our proposed method. Fig. 11 ▸ shows the log marginal likelihoods using the Gaussian, Matérn 3/2 and Matérn 5/2 kernels with various parameters. The log marginal likelihood has a single peak when *l* is varied and the other parameters are fixed, indicating that the log marginal likelihood is probably useful for selecting parameters.

**Figure 12:**
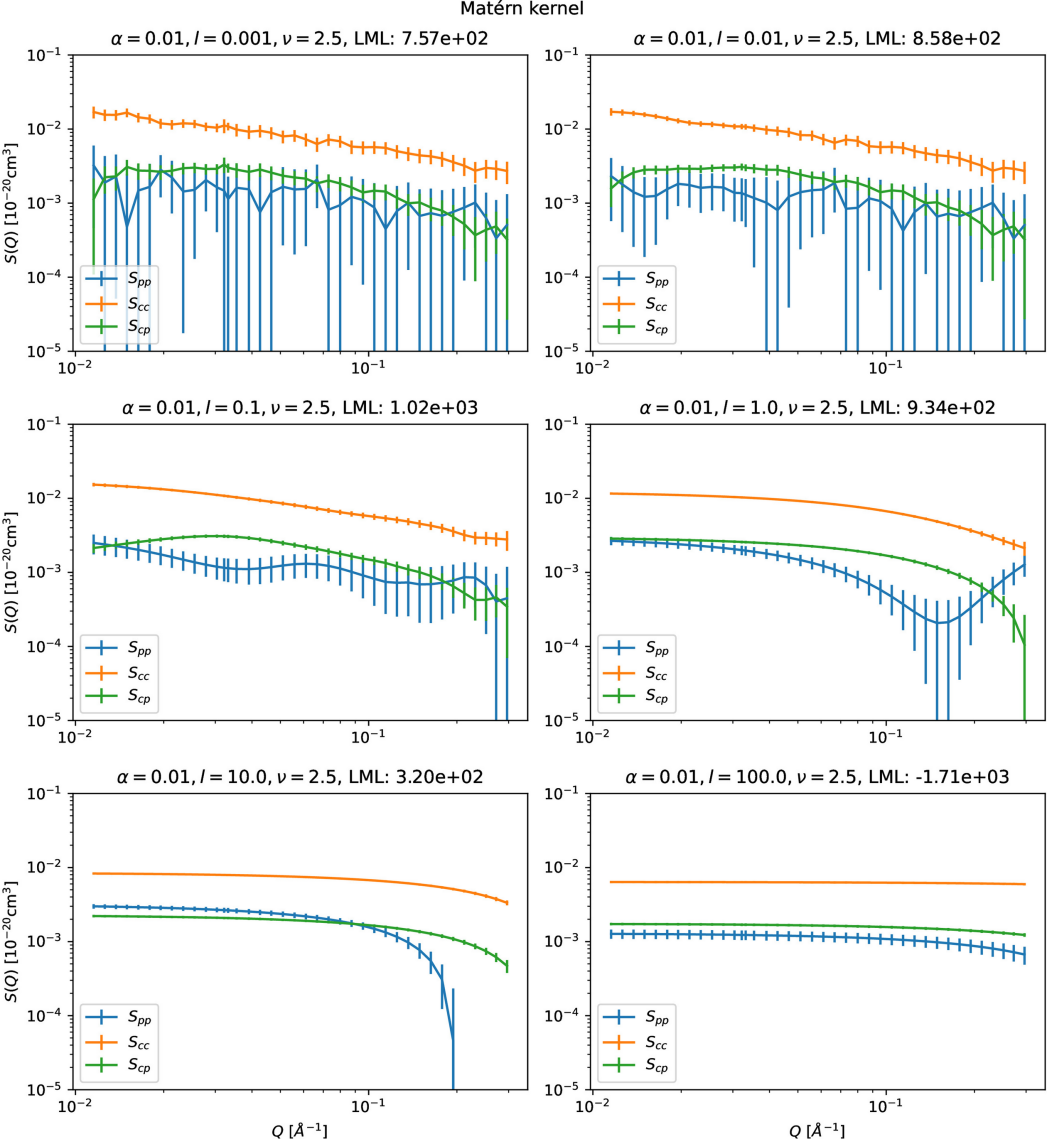
Partial scattering functions of polyrotaxane estimated with the Matérn 5/2 kernel with α = 0.01. LML stands for log marginal likelihood.

Fig. 12 ▸ shows the partial scattering functions estimated using the Matérn 5/2 kernel with α = 0.01. The following changes were observed when varying the scale parameter *l* from small to large:

(i) The estimated partial scattering functions for the smallest *l* look similar to that of weighted least squares.

(ii) As the scale parameters become larger, the estimated functions become less jagged, less bumpy and more monotonic. If the scale parameters are extremely large, the estimated functions become completely flat. The change is consistent with the meaning of the scale parameter.

(iii) As the scale parameters become larger, the error bars become smaller. This is because a large scale parameter requires a strong assumption about the flatness of the partial scattering function.

(iv) The relative errors within the high-*Q* range are large compared with those in the low-*Q* range. This reflects the large uncertainty in neutron intensities in the high-*Q* range.

The results for other kernels and parameters are shown in the supporting information. The findings are shared with the other kernels.

We also consider which parameters are appropriate in Fig. 12 ▸. The following points are important:

(i) The log marginal likelihood suggests that *l* = 0.1 is the best value.

(ii) The error bars for *l* ≤ 0.01 are too large to estimate the polyrotaxane structure reliably.

(iii) The curves are too flat for *l* ≥ 10. These curves appear to have lost important information about the structure.

The results overall show that *l* = 0.1 looks the best.

**Figure 13:**
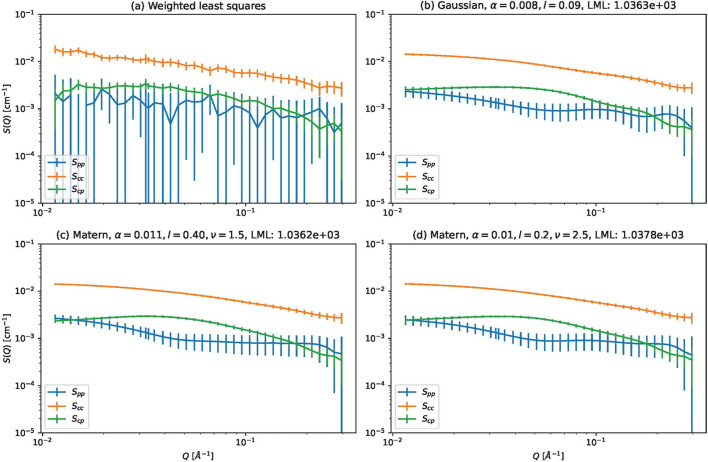
Comparison of cases for different kernels.

Next, we compare the effect of the kernel choice. Fig. 13 ▸ shows the estimated partial scattering functions using the Gaussian, Matérn 3/2 and Matérn 5/2 kernels with the parameters that give the maximum log marginal likelihood. We conducted a finer grid search to select the maximum log marginal likelihood.

From the result, we found the following points:

(i) The results look very similar and it is not clear which kernel is best.

(ii) The log marginal likelihoods are also very similar, and it is not useful to select kernels.

We now consider the phenomenon of the error bars shrinking for large *l*. The small error bars are due not only to an exact estimation but also to a strong assumption. Therefore, we cannot use the error bars to evaluate the parameters. It is also difficult to claim that the small error bars indicate an accurate estimate, since it is impossible to separate quantitatively the effect of an exact estimation from that of a strong assumption. In short, the parameter selection methods shown in Section 2.3 should be used.

## 4. Conclusion

In this paper, we have proposed a new method to estimate partial scattering functions from intensity functions using the idea of Gaussian process regression. The proposed method improves the estimated partial scattering functions by utilizing prior knowledge about their smoothness. The method also gives error bars since it uses Bayesian inference. Three types of parameter selection methods are also proposed.

The method was applied to synthetic core–shell and real polyrotaxane SANS data. The efficacy of the method is demonstrated in the applications. We have confirmed that the proposed method improves the estimation in some cases. We have also examined the choice of the kernel for the estimation.

We summarize the method on the basis of the findings of this paper in the following.

(i) Select a kernel. The Gaussian and Matérn kernels give similar results if we select appropriate parameters. Adding a small white kernel is important when the Gaussian kernel is used. If it is difficult to determine the weight of the white kernel, we use the Matérn kernel.

(ii) Select a kernel parameter selection method. The (*a*) subjective Bayesian approach, (*b*) subjective Bayesian approach using a hyper-prior and (*c*) empirical Bayesian approach are proposed in this paper. If we have sufficient prior knowledge about the experiment from a literature survey and preliminary experiments, (*a*) is recommended. If not, (*b*) or (*c*) is recommended. Even if we use (*b*) or (*c*), a literature survey or a preliminary experiment is recommended to determine the range of parameters.

(iii) Conduct an experiment and evaluate the scattering intensities and their errors. Appropriate error evaluation is important for estimating partial scattering functions. We note that overestimation of the errors is better than underestimation, as shown in Section 3.1.

(iv) Estimate partial scattering functions using the method introduced in Section 2.1 with the above kernel and the parameter selection method.

We discuss some extensions of the proposed method. First, we discuss the kernel functions. The kernel can reflect our prior knowledge of the partial scattering functions other than the smoothness. One example is introducing heterogeneity into the partial scattering functions. The Gaussian and Matérn kernels have the form *k*(*P*, *Q*) = φ(|*P* − *Q*|). This form represents the assumption that the partial scattering functions *S*(*Q*) have a similar smoothness and flatness for all *Q*. However, the assumption is not true in some cases, as shown in the example of CORE-SHELL-B. We can possibly represent the heterogeneity by modifying the kernel function.

Much of the literature, such as ch. 4 in Rasmussen and Willams’ textbook (Rasmussen & Williams, 2005 ▸) and Section 6.2 in Bishop’s textbook (Bishop, 2007 ▸), explains how to expand the kernel while keeping the symmetry and positive definiteness. Possible extensions of the Gaussian and Matérn kernels are shown as follows: 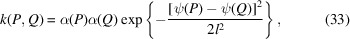

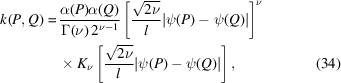
where ψ(*Q*) and α(*Q*) are functions. ψ(*Q*) can introduce the nonlinear change of coordinate on the *Q* axis and α(*Q*) can introduce a *Q*-dependent effect of the smoothness assumption. Further research is required on choosing functions ψ and α and how to extend the kernels.

The correlations between the partial scattering functions can possibly be represented in a kernel function. This study introduces no special knowledge about the relationship between the partial scattering functions. The covariant of the prior distribution, 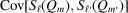, has the form 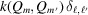 reflecting the information, where 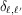 is the Kronecker delta. We can represent it in the kernel if we have prior knowledge of the relationship, such as the fact that two partial scattering functions are very similar. One way to introduce the relationship is changing 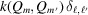 to 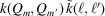, where 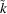 is another kernel to describe the relationship. To keep this paper brief, we merely introduce these ideas in Appendix *A*[App appa] and do not investigate them in detail.

Second, we consider the extension of the non-Gaussian prior distribution. Since the proposed method uses a multivariate Gaussian distribution as a prior distribution, the estimated partial scattering functions sometimes have negative values, but such negative values are not realistic. To reflect such prior knowledge, we can use a non-Gaussian distribution as a prior, such as log-normal or gamma distributions. A non-Gaussian prior makes estimation difficult. More complicated and expensive methods, such as variational methods or Markov-chain Monte Carlo, are required. Because of their complexity, we do not recommend non-Gaussian priors in normal cases. However, if we have important but unused prior knowledge about the experiment, such methods are worth considering.

This paper uses a simple grid search to select kernel parameters. We can refine the parameter search method using mathematical optimization such as the gradient method. Introducing such an optimization method into Gaussian process regression is discussed in Section 5.4.1 of the book by Rasmussen & Williams (2005 ▸), and we can use the technique for our proposed method.

The above ideas are out of this paper’s scope, and further research remains to be done.

## Supplementary Material

Additional figures. DOI: 10.1107/S1600576725003334/jl5111sup1.pdf

## Figures and Tables

**Figure 6:**
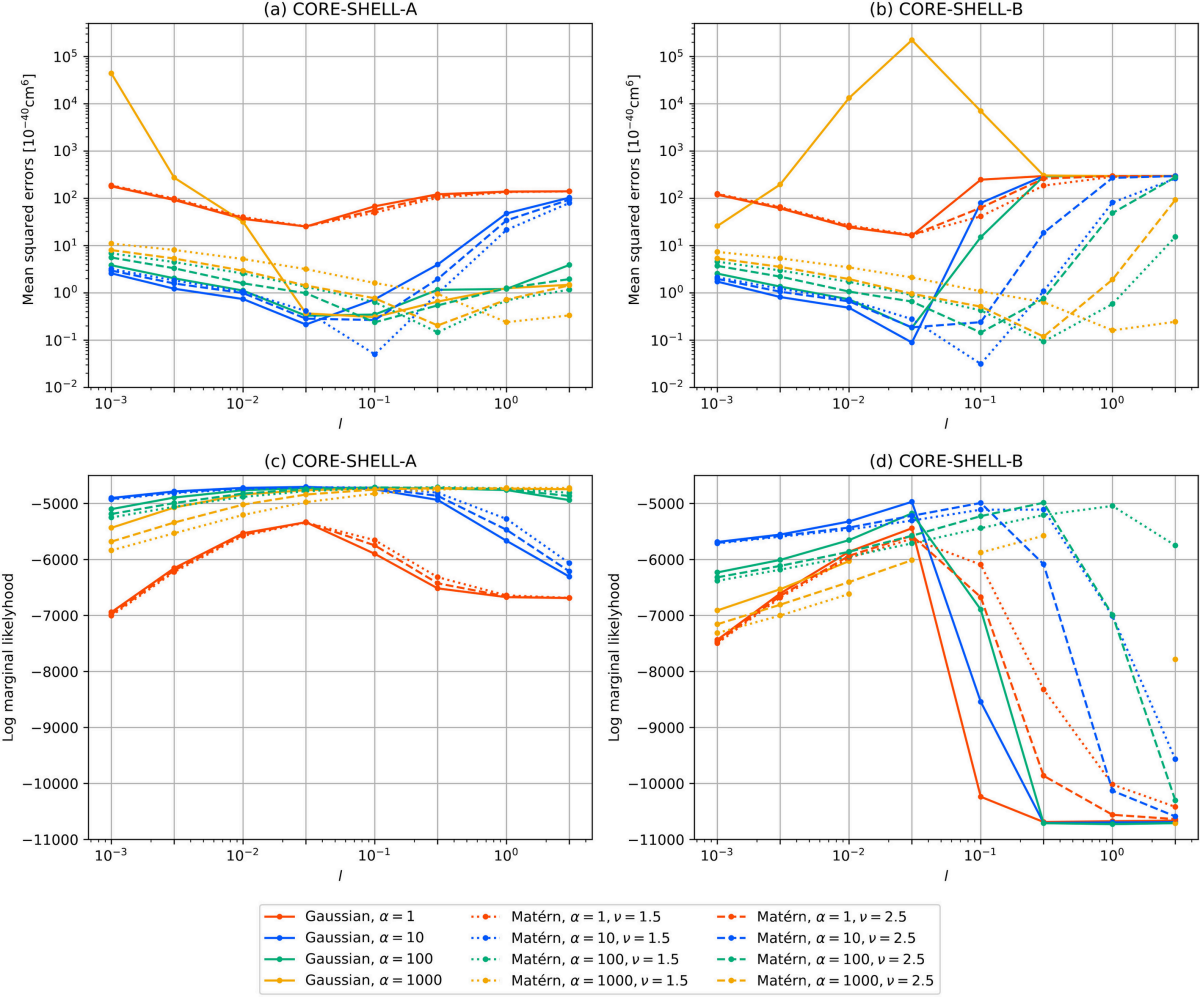
Mean squared errors and log marginal likelihoods for core–shell data with τ = 10^−5^. The mean squared errors were calculated using equation (32). (*a*) Mean squared errors between the estimated and expected partial scattering functions for CORE-SHELL-A. (*b*) Mean squared errors between the estimated and expected partial scattering functions for CORE-SHELL-B. (*c*) Log marginal likelihoods of the estimated partial scattering functions for CORE-SHELL-A. (*d*) Log marginal likelihoods of the estimated partial scattering functions for CORE-SHELL-B.

**Figure 14:**
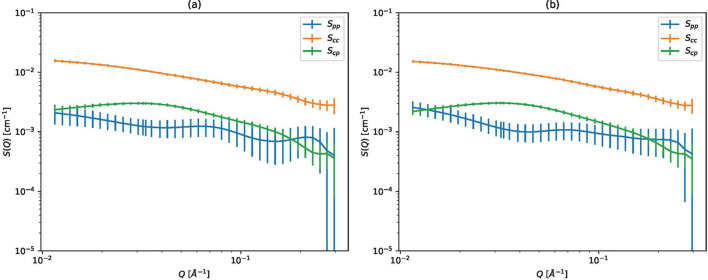
Estimated partial scattering functions of polyrotaxane using the kernel of equation (35), where *k* is the Matérn 5/2 kernel with α = 1.0 and *l* = 1.0, and (*a*) 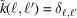 or (*b*) 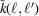 given by (36).

## Appendix A Extension of kernels

We here consider the possibility of extending the kernels. The following kernel is used for the polyrotaxane SANS data:

Since  should be positive definite, we introduce the following 3 × 3 matrix:

Fig. 14 ▸ shows the results, (*a*) without any assumption of correction among the three partial scattering functions and (*b*) with the assumption of correction between *S*_PP_ and *S*_CC_. The kernel parameters (θ_1_, θ_2_, θ_3_) are (0, 0, 0) for Fig. 14 ▸(*a*) and (π/3, 0, 0) for Fig. 14 ▸(*b*). *S*_PP_ and *S*_CC_ in Fig. 14 ▸(*b*) look more correlated than those of Fig. 14 ▸(*a*), as expected.

This result shows the flexibility of the proposed method. The question of extending the kernel and selecting parameters remains a subject for further research.
