# Supplementary material for: Enhanced estimation method for partial scattering functions in contrast variation small-angle neutron scattering via Gaussian process regression with prior knowledge of smoothness
Source: J Appl Crystallogr. 2025 May 31;58(Pt 3):976–91. doi: 10.1107/S1600576725003334 (PMC12135983; doi:10.1107/S1600576725003334)
Supplement: Supplementary file 1 [file j-58-00976-sup1.pdf]

JOURNAL OF APPLIED CRYSTALLOGRAPHY

Volume 58 (2025)

Supporting information for article:

Enhanced estimation method for partial scattering functions in contrast variation small-angle neutron scattering via Gaussian process regression with prior knowledge of smoothness

Ippei Obayashi, Shinya Miyajima, Kazuaki Tanaka and Koichi Mayumi

Section S1. Parameter search for polyrotaxane data

This section shows how the partial scattering functions of polyrotaxane are estimated by the proposed method using the Gaussian and Matérn kernels with various parameters. The ranges of parameters are as follows:

(i) α: 0.001, 0.01, 0.1, and 1.

(ii) l: 0.001, 0.01, 0.1, 1, 10, and 100.

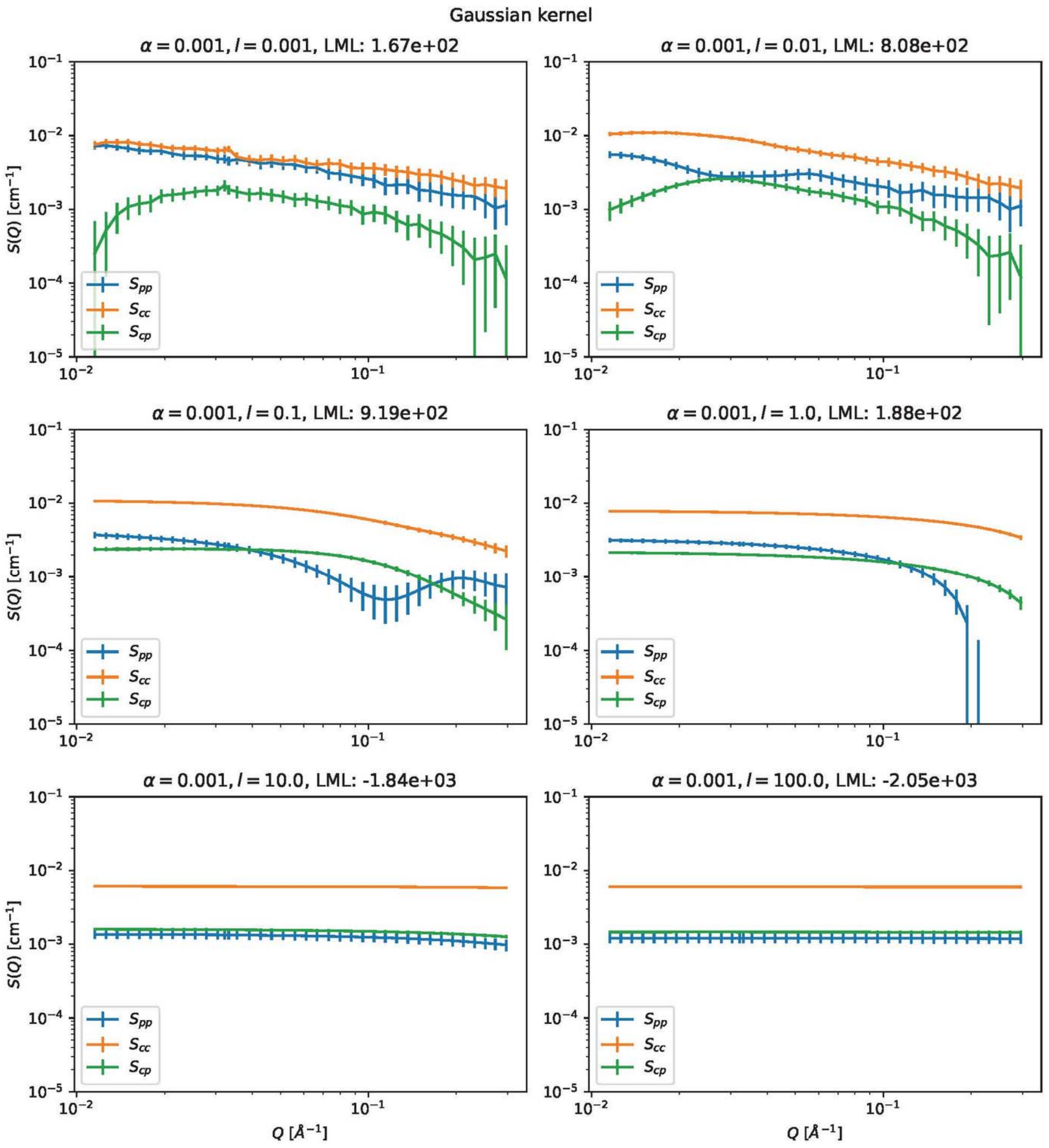

Fig. S1. Estimated partial scattering functions of polyrotaxane using the Gaussian kernel with α = 0.001.

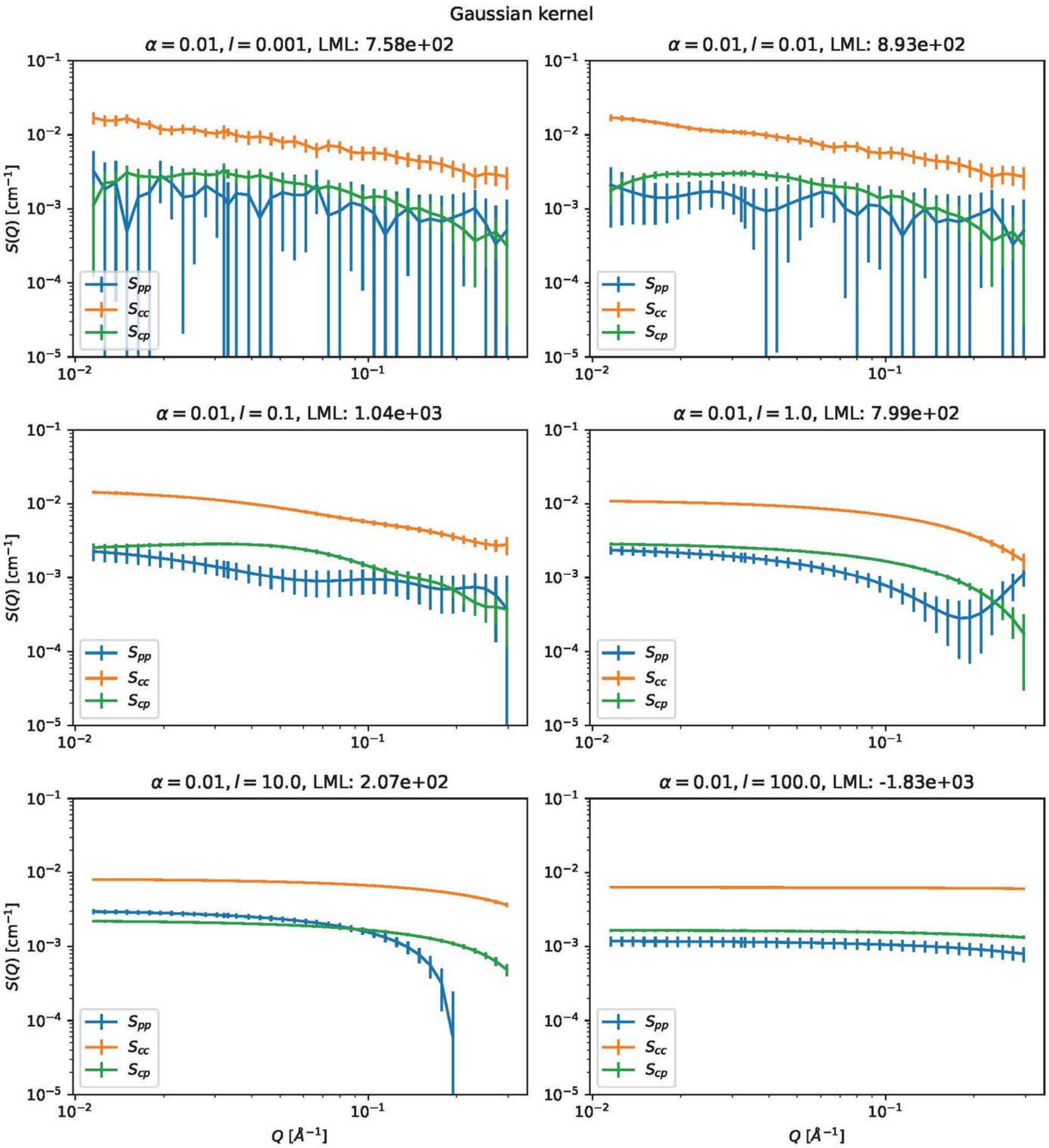

Fig. S2. Estimated partial scattering functions of polyrotaxane using the Gaussian kernel with α = 0.01.

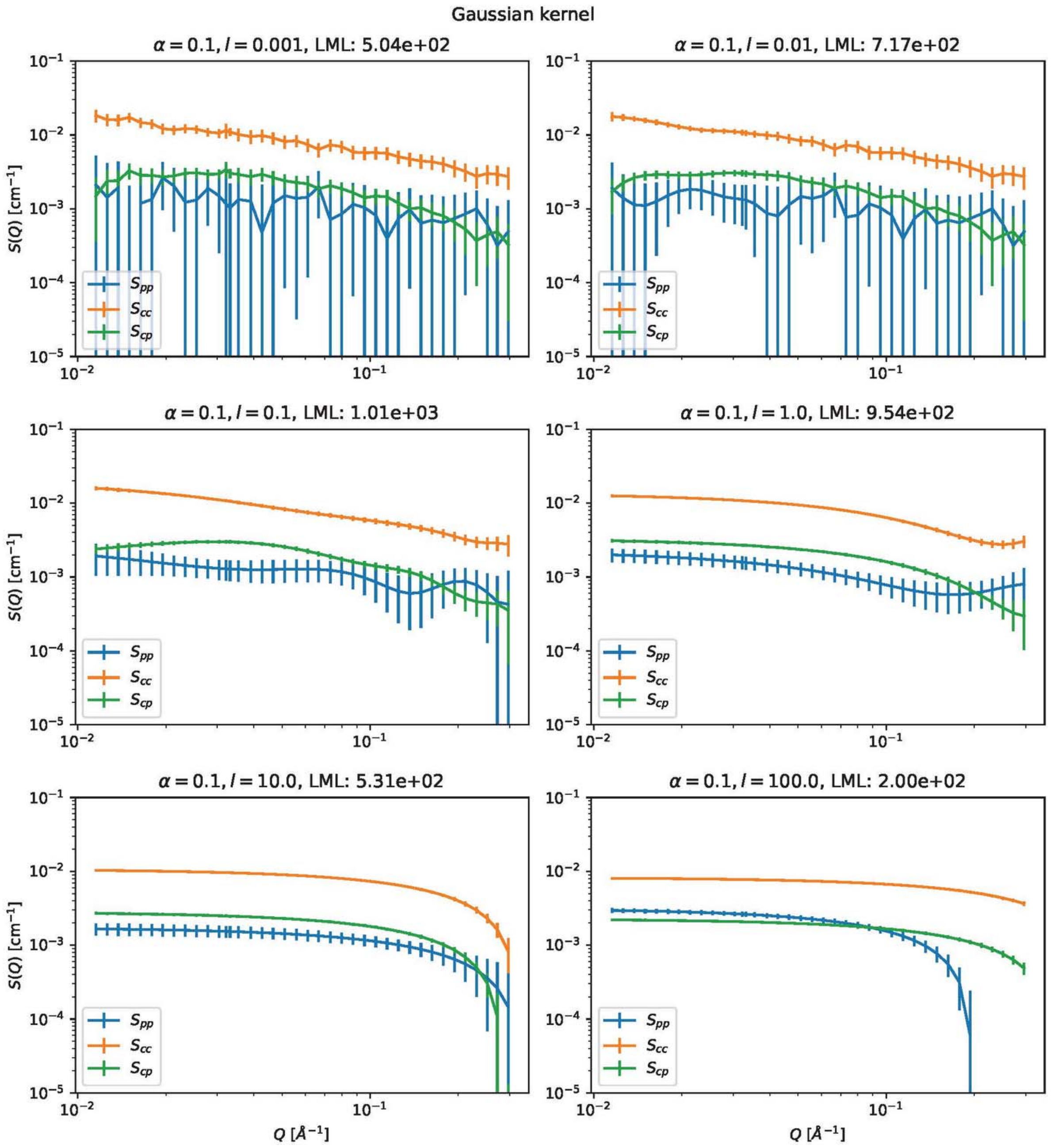

Fig. S3. Estimated partial scattering functions of polyrotaxane using the Gaussian kernel with α = 0.1.

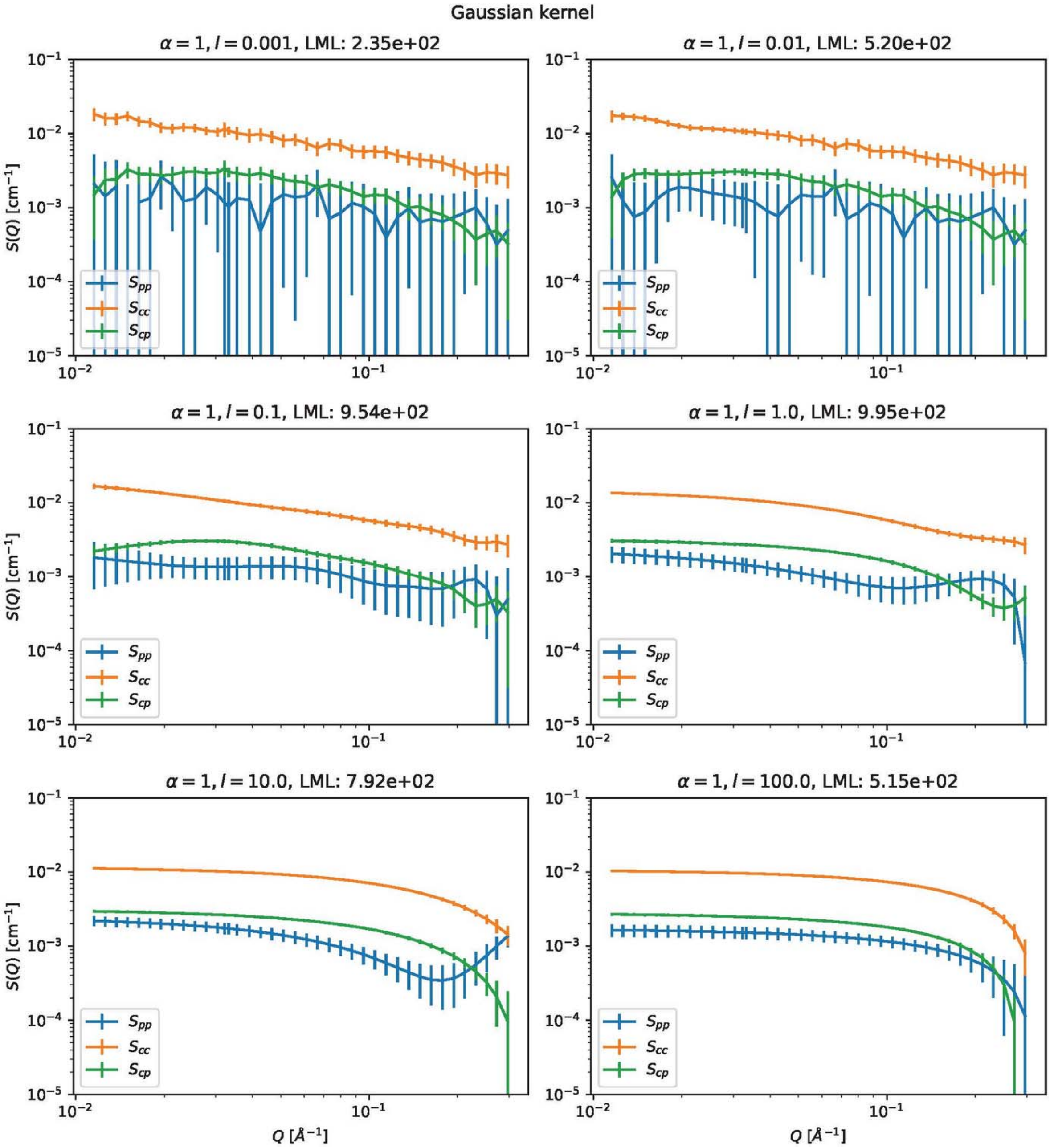

Fig. S4. Estimated partial scattering functions of polyrotaxane using the Gaussian kernel with α = 1.

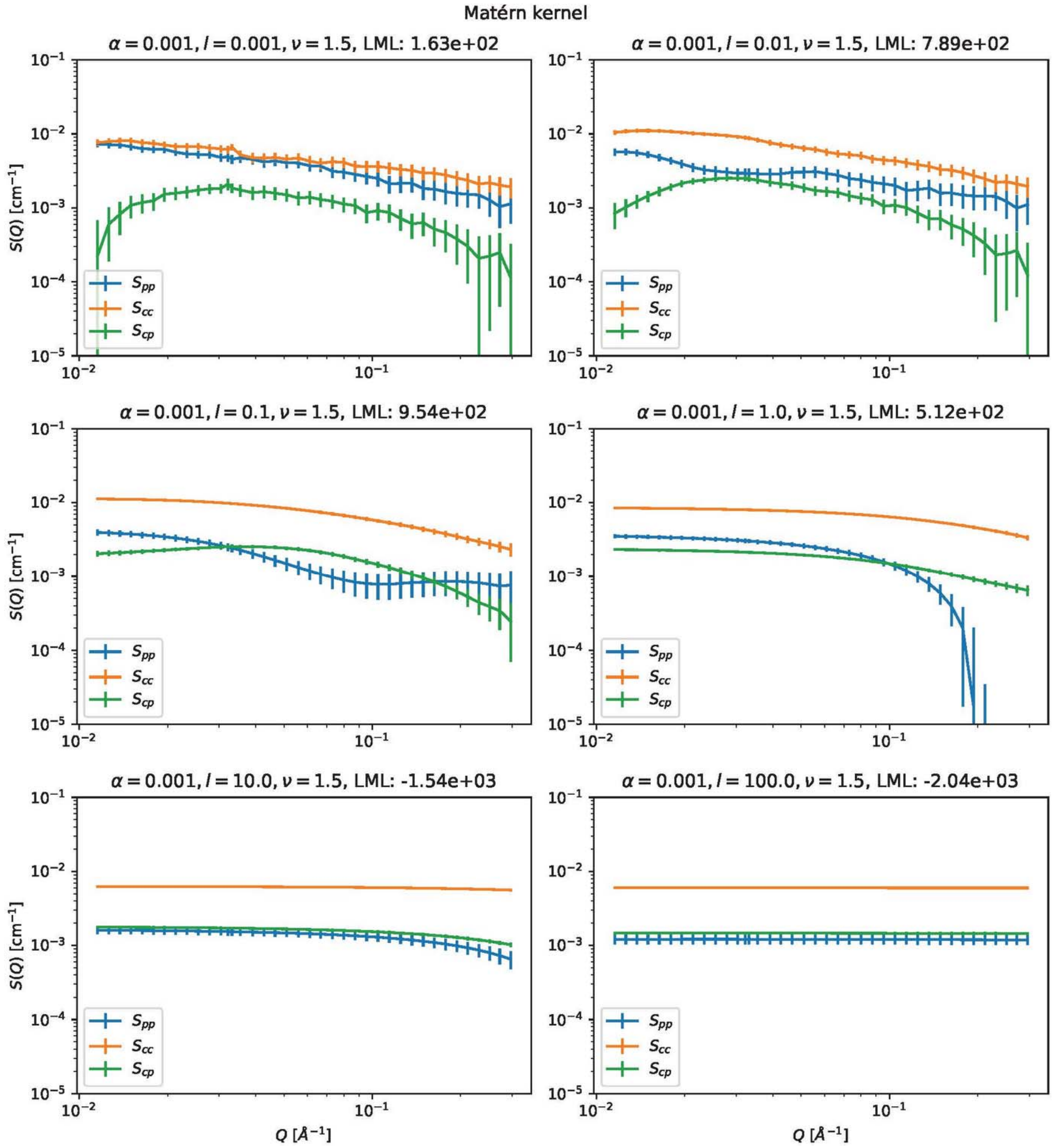

Fig. S5. Estimated partial scattering functions of polyrotaxane using the Matérn 3/2 kernel with α = 0.001.

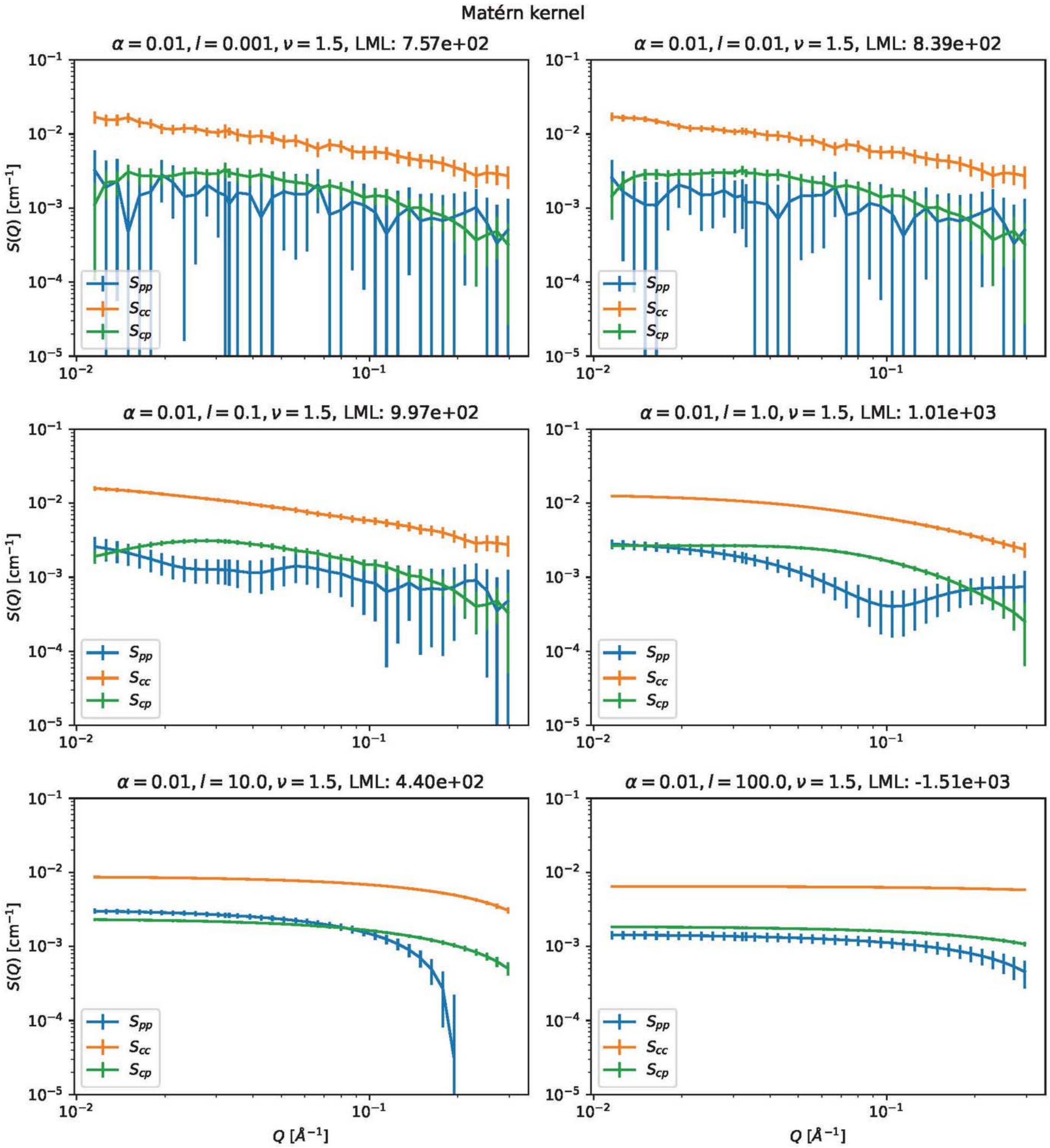

Fig. S6. Estimated partial scattering functions of polyrotaxane using the Matérn 3/2 kernel with α = 0.01.

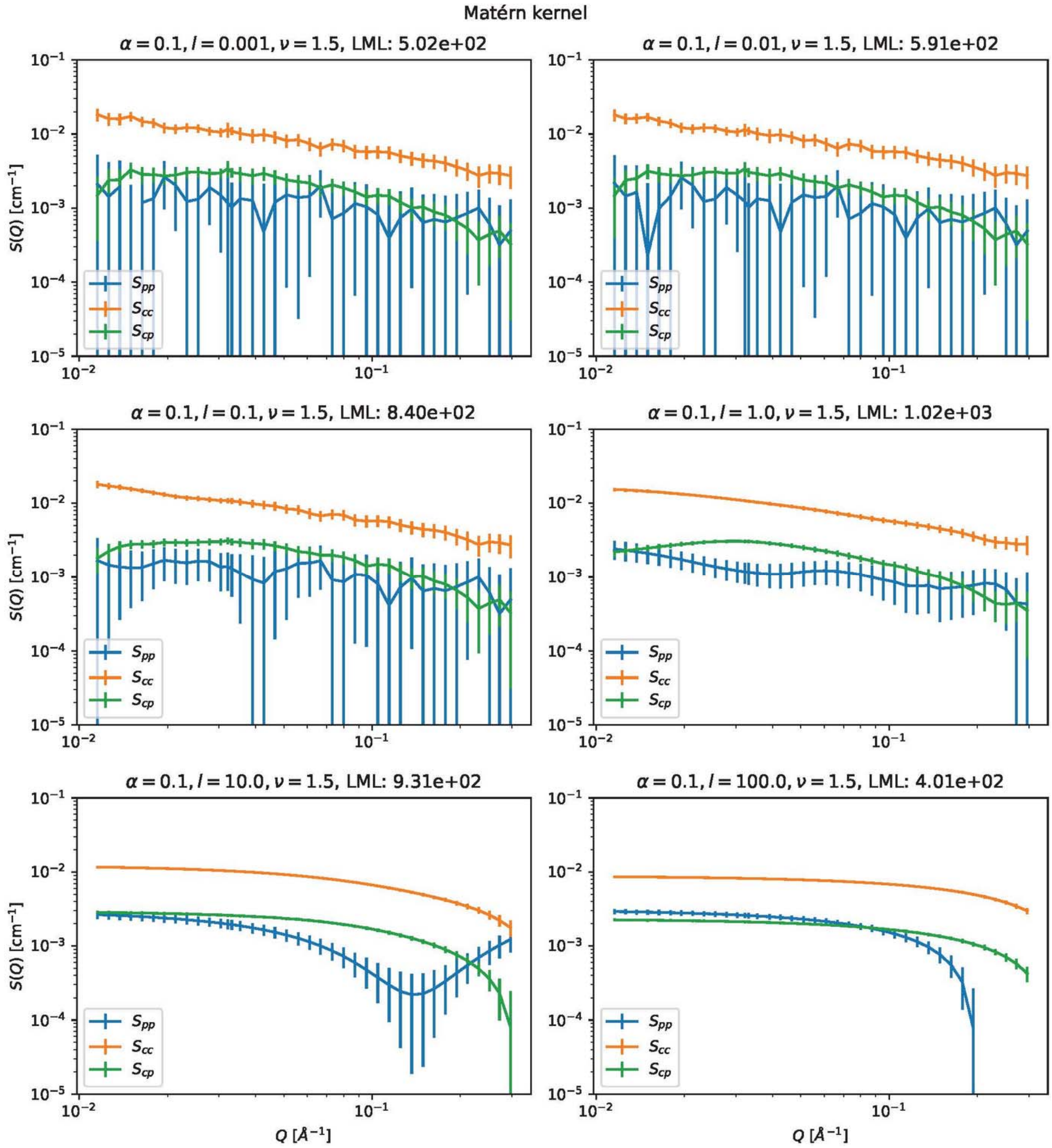

Fig. S7. Estimated partial scattering functions of polyrotaxane using the Matérn 3/2 kernel with α = 0.1.

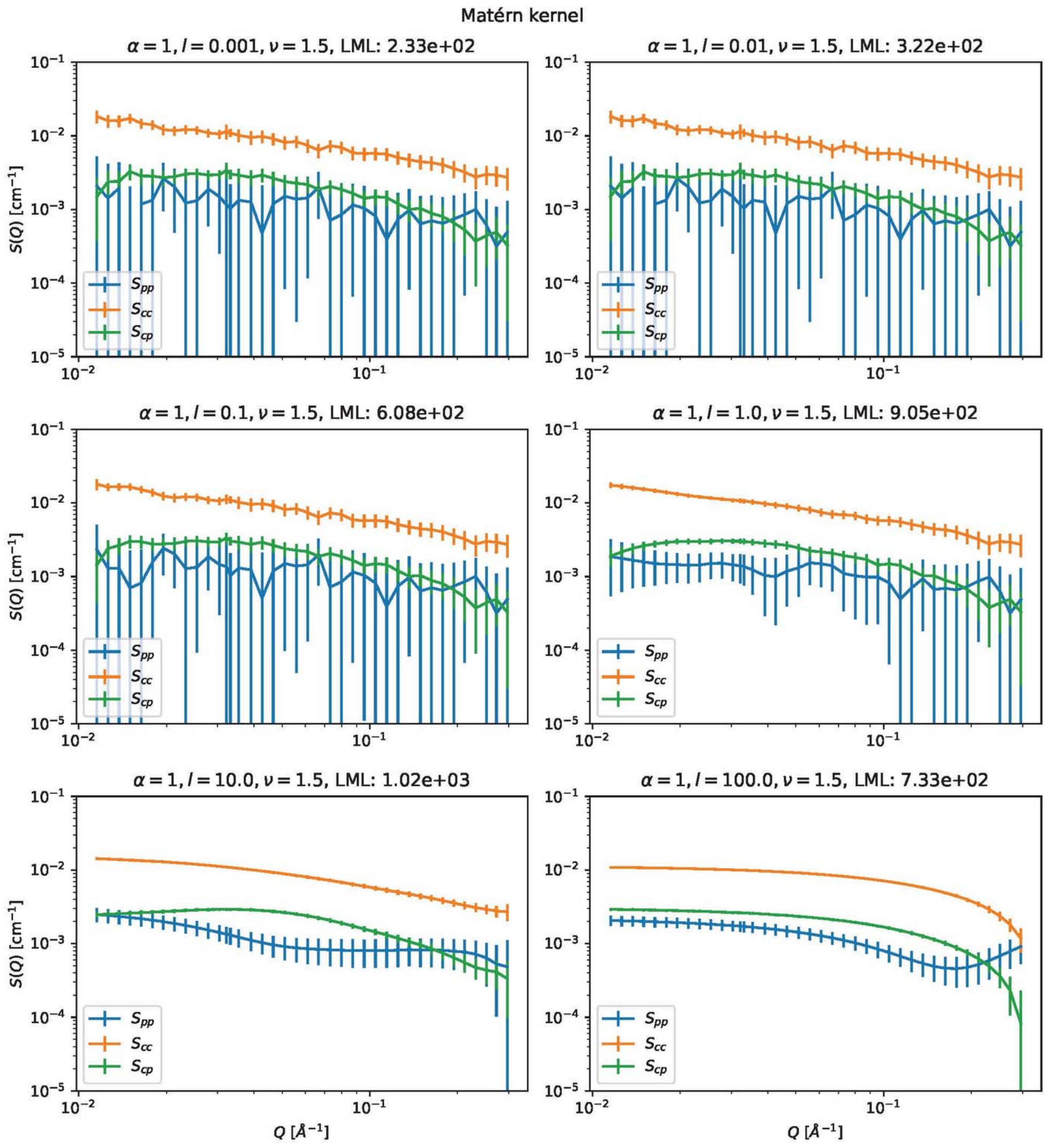

Fig. S8. Estimated partial scattering functions of polyrotaxane using the Matérn 3/2 kernel with α = 1.

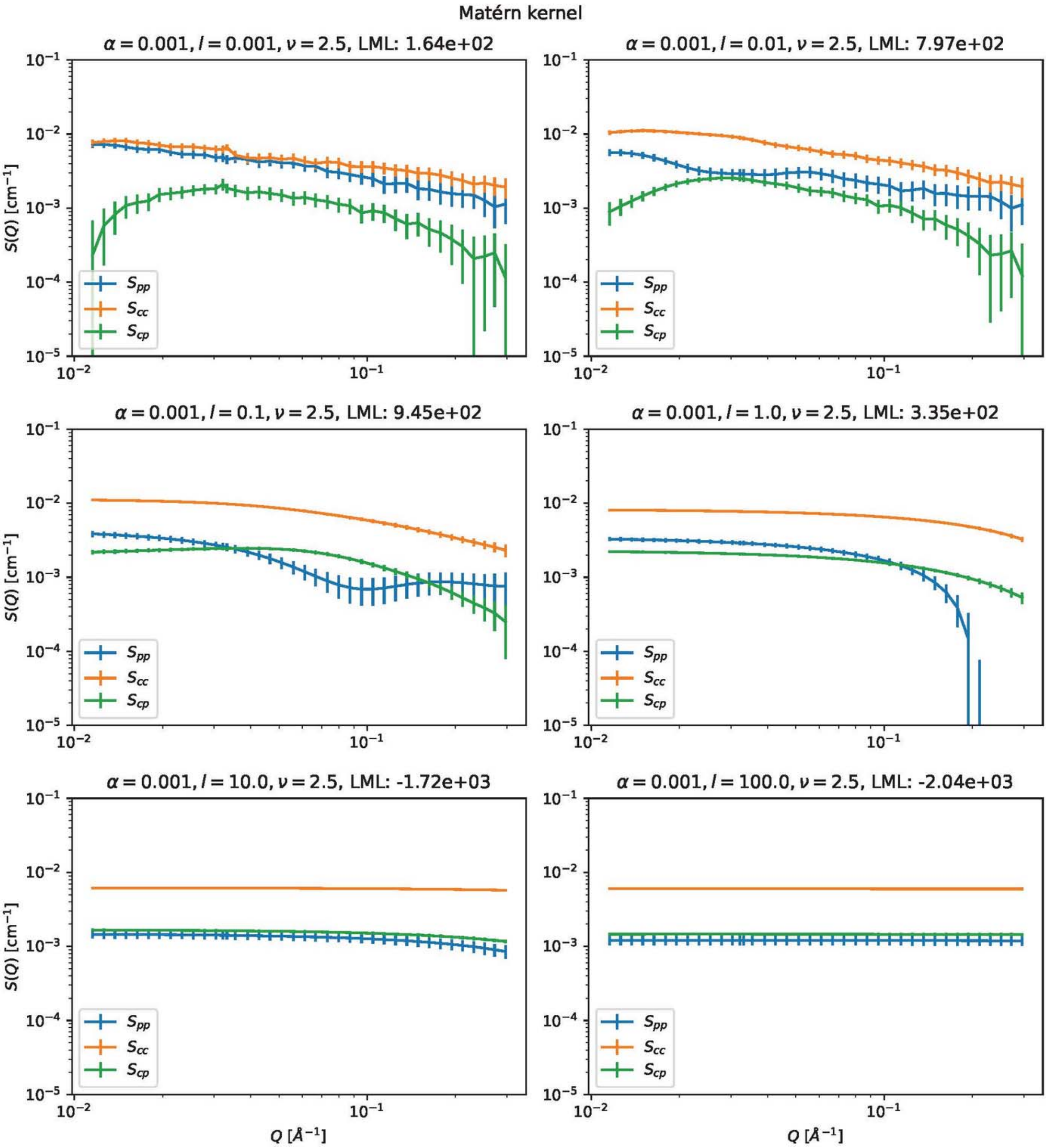

Fig. S9. Estimated partial scattering functions of polyrotaxane using the Matérn 5/2 kernel with α = 0.001.

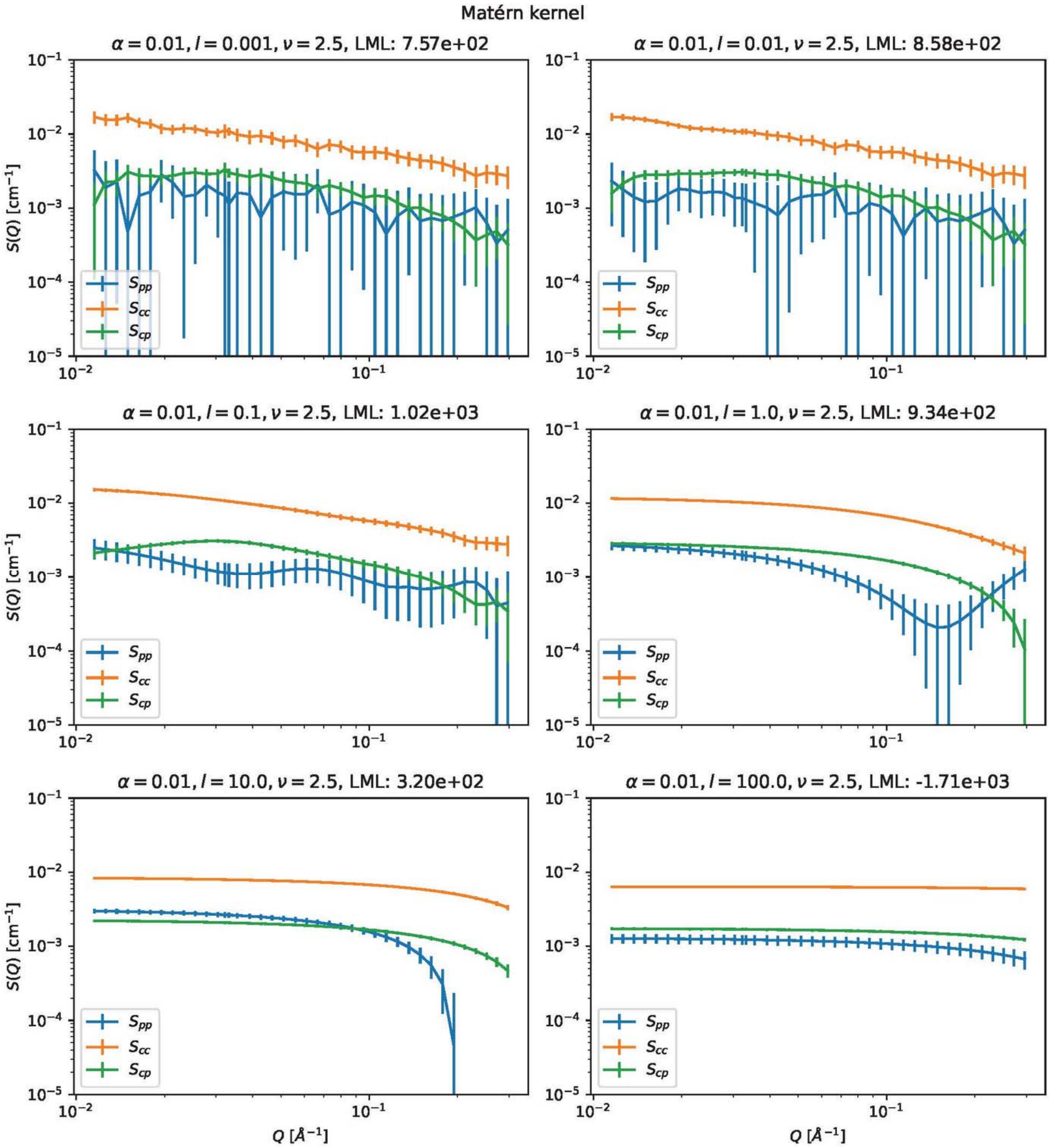

Fig. S10. Estimated partial scattering functions of polyrotaxane using the Matérn 5/2 kernel with α = 0.01.

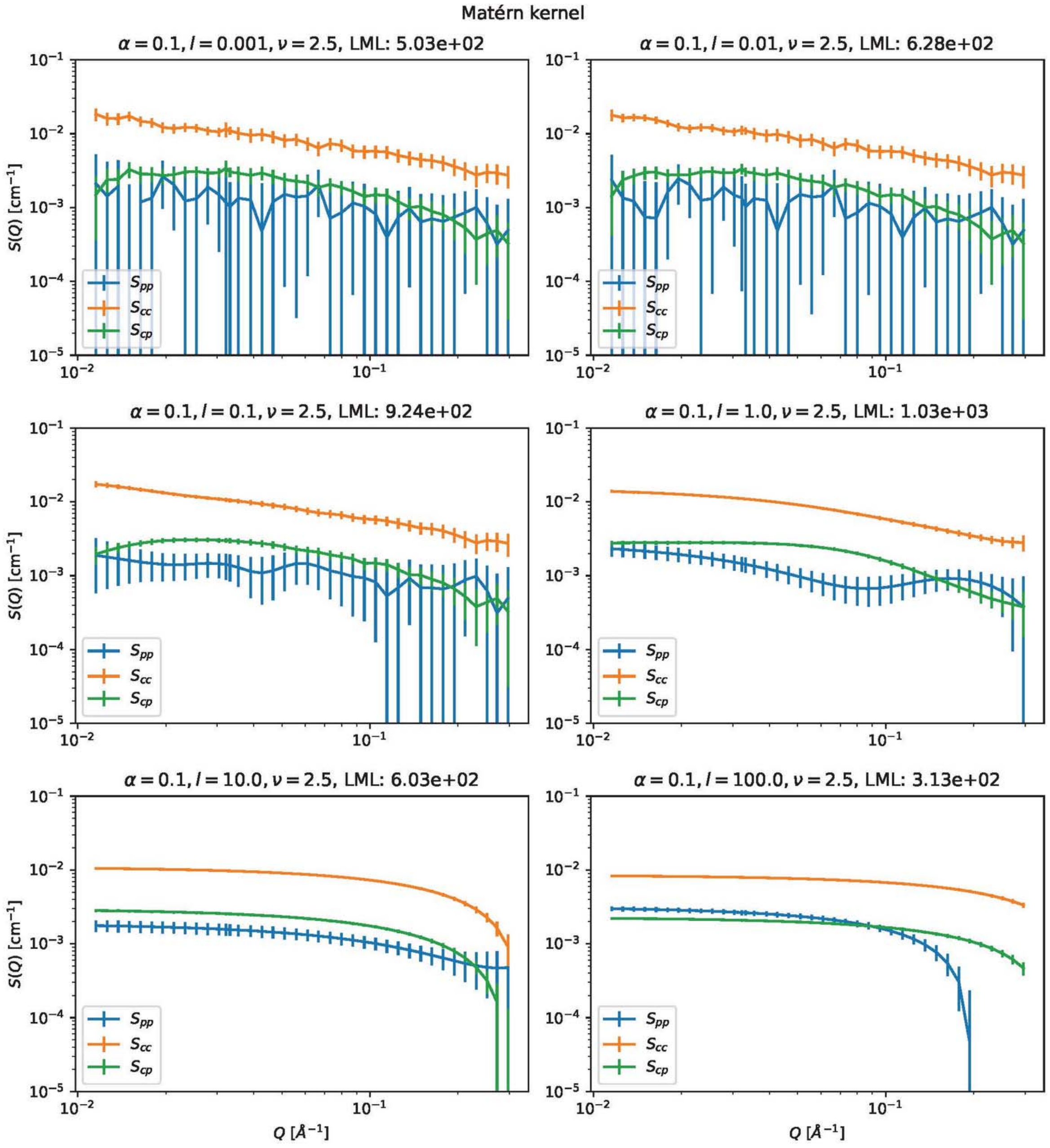

Fig. S11. Estimated partial scattering functions of polyrotaxane using the Matérn 5/2 kernel with α = 0.1.

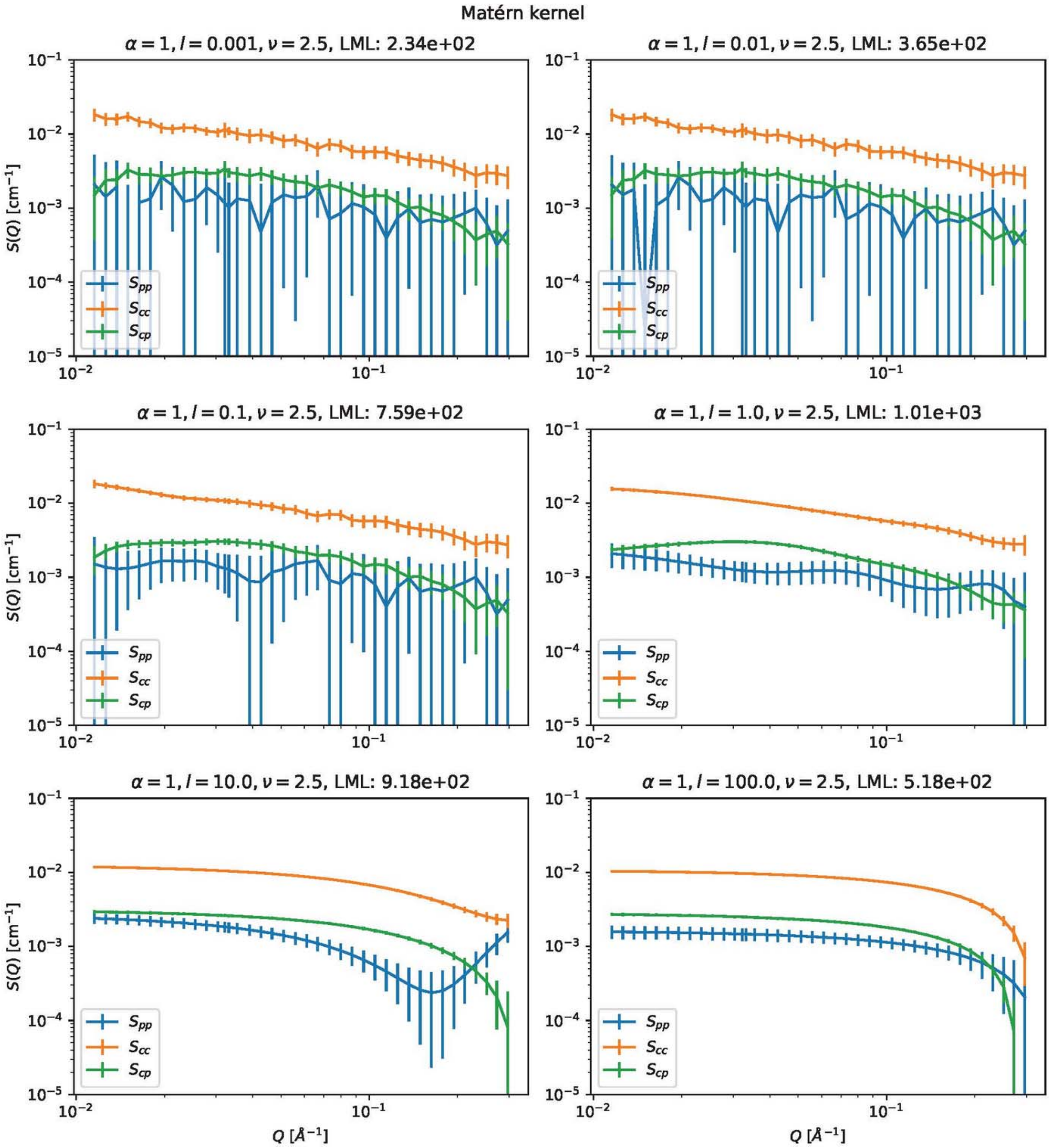

Fig. S12. Estimated partial scattering functions of polyrotaxane using the Matérn 5/2 kernel with α = 1.
